# Preconception cardiac rehabilitation: a structured narrative review and conceptual framework for maternal cardiovascular health and fetal growth

**DOI:** 10.3389/fmed.2026.1914342

**Published:** 2026-08-13

**Authors:** Jianxia Cheng, Fei Yu, Junsen Ye

**Affiliations:** First People's Hospital of Jiande City, Zhejiang, China

**Keywords:** cardiac rehabilitation, cardio-obstetrics, cardiovascular health, fetal growth restriction, narrative evidence mapping, preconception care, pregnancy complications

## Abstract

Cardiovascular disease is a major contributor to maternal morbidity and mortality, and many pregnancy-related cardiovascular deaths are potentially preventable. Cardio-obstetrics has advanced preconception counseling, multidisciplinary care, and risk stratification, but a structured preconception cardiovascular intervention model remains undeveloped. This structured narrative review synthesizes recent cardiovascular, exercise, lifestyle, and implementation evidence and proposes preconception cardiac rehabilitation (PCR) as a testable conceptual framework. Structured searches of PubMed, Scopus, Web of Science, Cochrane Library, CNKI, and Wanfang supported purposive selection of 39 key sources published from 2020 to 2026. A narrative evidence map organized the curated literature by reproductive phase, intervention type, outcome domain, and evidence level; it was not designed as a formal systematic evidence gap map. No study directly evaluated integrated PCR. Indirect evidence suggests that preconception physical activity and cardiorespiratory fitness may be associated with lower risks of several adverse pregnancy outcomes, whereas evidence for fetal growth restriction remains particularly limited. PCR is therefore presented as cardiovascular prehabilitation for pregnancy, not as a validated care model. The revised framework specifies candidate eligibility groups, referral routes, responsible disciplines, follow-up points, implementation barriers, and equity considerations. Consensus development, feasibility studies, cost and implementation evaluations, and randomized trials are needed before clinical adoption.

## 1 Introduction

Cardiovascular disease (CVD) is one of the most important contributors to maternal morbidity and mortality. Contemporary cardio-obstetric literature emphasizes that many pregnancy-related cardiovascular deaths are preventable, yet affected women are often not recognized as high risk before pregnancy (1–4). This gap between preventability and late recognition makes the preconception period a critical window for intervention.

The biological rationale for preconception cardiovascular optimization is compelling. Adverse pregnancy outcomes (APOs), such as hypertensive disorders of pregnancy (HDP), preeclampsia, gestational diabetes mellitus (GDM), preterm birth, small-for-gestational-age birth, and fetal growth restriction (FGR), are not events that emerge only after mid-pregnancy. Many vascular, metabolic, inflammatory, and placental pathways begin before conception or during very early gestation (1, 5–7). By the time pregnancy is clinically recognized, early placental development and maternal cardiovascular adaptation may already be underway. Interventions initiated only after pregnancy confirmation may therefore be biologically late.

Two mature bodies of clinical knowledge remain insufficiently connected. Cardiac rehabilitation (CR) is an established, structured, multicomponent intervention for cardiovascular prevention and recovery. The 2024 update from the American Heart Association (AHA) and the American Association of Cardiovascular and Pulmonary Rehabilitation defines core CR components, such as patient assessment, nutritional counseling, body composition management, cardiovascular risk factor management, psychosocial care, aerobic exercise training, strength training, physical activity counseling, and program quality (8). Cardio-obstetrics has developed models for preconception counseling, pregnancy heart team care, and risk stratification using the modified World Health Organization (mWHO) classification, CARPREG II, and ZAHARA (3, 4, 9–12). However, it has not translated CR into a defined preconception intervention pathway.

The primary purpose of this structured narrative review is to synthesize evidence relevant to preconception cardiovascular optimization and use that synthesis to propose PCR as a testable conceptual framework. A narrative evidence map is used as a secondary organizing tool to show the distribution of the curated literature; it is not intended to constitute a formal systematic evidence gap map or a clinical guideline.

This review addressed three questions:

How can the 2024 AHA/AACVPR CR core components be adapted to preconception care?

What is the current distribution and quality of evidence for cardiovascular, exercise, and lifestyle interventions across the preconception, pregnancy, and postpartum continuum?

What are the highest-priority research gaps for evaluating PCR, particularly regarding maternal complications and FGR?

## 2 Methods

### 2.1 Study design

We conducted a structured narrative review to support development of a conceptual PCR framework. A narrative evidence-mapping exercise was embedded as a secondary descriptive component. Sources were identified through structured database searches and expert knowledge, then purposively curated for relevance, directness, and authority. Priority was given to professional society guidance, systematic reviews, meta-analyses, directly relevant cohort studies, intervention studies, and cardio-obstetric pathway papers. As screening was not exhaustive or performed independently by two reviewers, and formal risk-of-bias tools were not applied, this work does not meet methodological standards for a formal evidence gap map, systematic review, or PRISMA-ScR-compliant scoping review.

### 2.2 Search strategy

Searches were designed for PubMed and adapted for Scopus, Web of Science, Cochrane Library, CNKI, and Wanfang. The search period was January 2020 to June 2026. This period was selected to capture the recent development of cardio-obstetrics as a structured field, the 2023 AHA statement on prepregnancy cardiovascular health, and the 2024 AHA/AACVPR update of CR core components (1, 8).

Search concepts included preconception or prepregnancy care AND exercise or cardiac rehabilitation AND pregnancy outcomes, preconception cardiovascular health or risk factor management AND adverse pregnancy outcomes, cardio-obstetrics or pregnancy heart team AND preconception care, CR core components, exercise prescription, and program quality, postpartum cardiovascular risk reduction after APOs, preconception cardiorespiratory fitness, hemodynamics, placental function, and FGR.

The full search strategies are provided in Supplementary File 1. Core PubMed searches combined MeSH and free-text terms for preconception care, cardiac rehabilitation, exercise, cardiovascular risk factors, and pregnancy outcomes, with date and language filters. Records identified across the six databases were reviewed for relevance to the framework, but exported search records were not maintained as a single deduplicated screening set. Therefore, total database yield, deduplicated yield, and stage-specific exclusion counts cannot be reported reliably. The final curated corpus comprised 39 key sources selected according to the priority hierarchy in Section 2.1. This number should not be interpreted as the yield of a systematic screening process.

To improve transparency, source selection followed four sequential questions: whether the source addressed preconception cardiovascular health or CR-adaptable components; whether it reported or synthesized maternal, fetal, neonatal, or postpartum cardiovascular outcomes; whether it had sufficient methodological detail for interpretation; and whether it added information not already covered by a higher-level or more directly relevant source.

Sources were excluded during curation when they were mechanistically remote from PCR, addressed preconception care without cardiovascular relevance, had insufficient outcome information, or duplicated evidence already represented by a more authoritative synthesis.

### 2.3 Eligibility criteria

Sources were eligible if they addressed at least one of the following domains: preconception cardiovascular health, CR components relevant to preconception adaptation, cardio-obstetric preconception care, exercise, diet, or lifestyle interventions before or during pregnancy, postpartum cardiovascular prevention after APOs, or mechanisms linking maternal cardiovascular physiology with fetal growth.

We excluded non-human studies, case reports, non-cardiovascular preconception interventions without relevance to the framework, conference abstracts without sufficient data, and studies with no maternal or fetal outcome relevance.

### 2.4 Evidence classification and appraisal

Evidence was classified using a seven-level hierarchy. Systematic reviews, meta-analyses, scientific statements, and reporting guidelines were treated as Level I evidence, randomized controlled trials (RCTs) as Level II, cohort and case–control studies as Level IV, and narrative reviews, protocols, and pathway papers as Level V. Classification was based on study design as reported in each source. Formal risk-of-bias appraisal (AMSTAR-2, RoB 2, ROBINS-I, AGREE II) was not performed, consistent with the purposive rather than systematic evidence synthesis approach (see Section 2.1).

Evidence quality was therefore interpreted cautiously using directness, consistency, population applicability, and study-design hierarchy rather than formal certainty ratings. Claims were treated as direct evidence only when a source evaluated the relevant exposure, intervention, population, and outcome. Evidence extrapolated from conventional CR, general preconception lifestyle interventions, pregnancy exercise trials, or postpartum cardiovascular prevention was labeled as indirect evidence or expert proposal.

This distinction is important for PCR because the strongest evidence supports the plausibility of cardiovascular optimization, whereas no source yet demonstrates that an integrated PCR program improves maternal or fetal outcomes.

### 2.5 Narrative evidence-mapping approach

The narrative evidence map used a descriptive matrix with intervention type, outcome domain, and reproductive phase as organizing dimensions. Categories were defined before synthesis and applied to the 39-source curated corpus. Evidence density was assigned qualitatively from the number, design, directness, and maturity of sources in each cell. No duplicate coding, formal certainty assessment, or risk-of-bias appraisal was undertaken. Accordingly, the map supports conceptual interpretation and gap identification but does not provide the completeness or reproducibility of a formal evidence gap map (39).

No PRISMA flow diagram is presented because the review did not use systematic duplicate screening from a stable record set. We instead report the curated source categories and explicitly acknowledge this as a methodological limitation. The map should be read as a conceptual synthesis tool, not as a complete inventory of all eligible studies.

## 3 Results

### 3.1 Characteristics of the curated source corpus

Thirty-nine key sources informed the working evidence corpus through purposive, curated selection (see Section 2.1 for methodological rationale). These included AHA and European scientific statements (*n* = 6), systematic reviews and meta-analyses (*n* = 14), cardio-obstetrics narrative reviews and pathway papers (*n* = 5), evidence-mapping methodology papers (*n* = 3), pregnancy and postpartum intervention reviews (*n* = 6), and mechanistic studies linking maternal cardiovascular physiology with pregnancy outcomes (*n* = 5). Approximately two-thirds of sources were classified as Level I evidence (systematic reviews, meta-analyses, or scientific statements from major professional societies). All sources were drawn from indexed, peer-reviewed journals or professional society statements. The reference list spans 2020 to 2026, a period that captures the recent consolidation of cardio-obstetrics as a named field and the 2023–2024 cycle of AHA scientific statements on prepregnancy cardiovascular health and CR core components.

The main reason for retaining a source was its contribution to one of the four framework needs defining the preconception cardiovascular problem, adapting CR components to a reproductive context, identifying maternal or fetal outcomes that could be evaluated in PCR studies, or informing delivery and follow-up. Sources that were broader than these needs were used only when they supplied high-level guidance or implementation principles.

Geographically, the evidence base is concentrated in high-income countries. Most included reviews drawn predominantly from North American, European, and Australian cohorts. LMIC-specific evidence was limited to a small number of reviews addressing preconception care in South Asian and sub-Saharan African settings. The CNKI/Wanfang search identified Chinese-language studies that remain largely descriptive or protocol-level. This geographic asymmetry is a structural limitation of the current evidence, not merely a reporting gap. Populations at greatest risk of undetected cardiovascular disease in pregnancy may also have the least preconception research infrastructure. However, this inference has not been systematically verified and should be treated as an expert observation rather than a formal finding.

The temporal distribution reinforces the preconception gap. Pregnancy-phase intervention trials and systematic reviews have accumulated steadily since 2020, with several large meta-analyses published in 2023–2025. Postpartum cardiovascular prevention research has accelerated following the 2024 AHA postpartum scientific statement. Preconception-phase intervention evidence, in contrast, has grown slowly. The number of eligible preconception RCTs identified across all included reviews was small, and most evaluated broad health promotion rather than structured cardiovascular intervention.

### 3.2 Main evidence pattern: the preconception evidence cliff

The clearest pattern in the curated literature was a marked reduction in intervention evidence before conception compared with pregnancy.

Pregnancy-phase exercise and lifestyle interventions have been evaluated extensively. Multiple systematic reviews and meta-analyses, encompassing dozens of RCTs and thousands of participants, have examined aerobic exercise, combined diet-and-exercise interventions, and, to a lesser extent, resistance training during pregnancy (13, 14). These reviews generally report favorable or neutral effects on gestational weight gain, GDM risk, and blood pressure, with no signal of harm for appropriately prescribed exercise in uncomplicated pregnancies.

The preconception evidence base is thinner by an order of magnitude. Existing preconception lifestyle reviews identify only a small number of RCTs that began interventions before pregnancy and continued to measure pregnancy outcomes (15–18). Most of these trials evaluated broad diet or physical activity counseling rather than a structured, multicomponent cardiovascular optimization protocol. Key design features of CR (individualized exercise prescription guided by functional testing, multicomponent delivery, standardized quality metrics, and longitudinal outcome tracking) are absent from the existing preconception trial literature.

No identified study evaluated an integrated PCR intervention combining cardiovascular assessment, individualized exercise, nutrition, risk-factor optimization, psychosocial care, and quality monitoring. The gap therefore concerns the absence of a tested multicomponent model rather than the absence of the term PCR.

### 3.3 Evidence for preconception physical activity and cardiorespiratory fitness

Physical activity: Available evidence suggests that higher preconception physical activity, particularly moderate-to-vigorous physical activity (MVPA), may be associated with lower risks of HDP, preeclampsia, GDM, excessive gestational weight gain, and low birth weight (15, 17, 18). Murrell and Joseph conducted an integrative review of preconception physical activity studies published from 2016 to 2025 and reported that higher preconception MVPA was generally associated with improved maternal health outcomes, but the number of eligible studies was small, exposure measurement was heterogeneous (self-report in most studies, device-based in a minority), and prospective studies with pregnancy outcome follow-up were rare (17). A systematic review and meta-analysis of pre-pregnancy diet and physical activity interventions for GDM prevention found suggestive but inconclusive evidence, with substantial heterogeneity across intervention designs and populations (16).

Cardiorespiratory fitness: Al-Huda and colleagues conducted the first systematic review and meta-analysis of the association between preconception CRF and HDP. Across a small number of studies, they found that lower CRF was associated with increased HDP risk, with a suggested threshold of VO_2max_ below approximately 37 mL/kg/min (15). The evidence base was limited by small aggregate sample size, heterogeneous fitness measures (VO_2max_ estimated from submaximal testing, directly measured VO_2max_, and self-reported physical activity as a proxy), and incomplete adjustment for confounders including prepregnancy BMI. The direction of association is consistent with general population data linking CRF to cardiovascular outcomes, but the specific evidence for preconception CRF as an independent predictor of HDP remains preliminary.

Dose–response and biological gradient: The relationship between prepregnancy BMI and HDP risk provides indirect support for cardiovascular optimization. A dose–response meta-analysis of 157 cohort studies involving over 50 million pregnancies found that each 5 kg/m^2^ increase in prepregnancy BMI was associated with a 75% higher risk of HDP (adjusted OR 1.75, 95% CI 1.68–1.82), with stronger associations in Asian populations (19). An individual participant data meta-analysis of 39 cohorts estimated that 23.9% of pregnancy complications were attributable to maternal overweight or obesity (20). An umbrella review of 53 systematic reviews concluded with moderate certainty that prepregnancy physical activity, BMI, and interpregnancy weight change are important modifiable risk factors for adverse pregnancy outcomes (21). The NHSII prospective cohort study found that six prepregnancy healthy lifestyle factors, including healthy BMI and regular physical activity, were associated with a 37% lower risk of adverse pregnancy outcomes (RR 0.63, 95% CI 0.55–0.72). It is estimated that 19% of adverse outcomes might be preventable through preconception lifestyle modification (22).

Together, these findings support the biological plausibility of preconception cardiovascular optimization. However, they do not establish that a structured multicomponent program reduces HDP or other pregnancy endpoints.

### 3.4 Evidence for fetal growth restriction: hemodynamic and placental pathways

Direct evidence linking preconception cardiovascular optimization to FGR is particularly limited.

Hemodynamic phenotype before pregnancy. The CONCEIVE Study, a prospective longitudinal study of 387 women followed from preconception through postpartum, found that women who later developed preeclampsia or FGR had a distinct preconception hemodynamic phenotype. This included lower cardiac output, higher total peripheral resistance, and altered responses to exercise stress testing, with a larger reduction in total peripheral resistance and a smaller increase in stroke volume during exercise (23). These findings suggest that subclinical cardiovascular dysfunction may be detectable before pregnancy and may be identified through preconception cardiopulmonary exercise testing (CPET).

The PEACH study (*n* = 906) subsequently confirmed that the clinical relevance of third-trimester hemodynamics low cardiac output (<6.0 L/min) combined with FGR was associated with a fivefold higher risk of preterm preeclampsia (HR 5.25, 95% CI 1.26–21.9), and high systemic vascular resistance (≥ 1,200 dynes·s·cm^−5^) was strongly associated with all forms of HDP (24). Whether preconception hemodynamic optimization through exercise training alters these trajectories is unknown.

Placental and vascular mechanisms: Mechanistically, aerobic exercise enhances endothelial function, placental angiogenesis, and plasma volume expansion through shear-stress-mediated endothelial nitric oxide synthase (eNOS) phosphorylation and vascular endothelial growth factor (VEGF) upregulation (25, 26). Maternal low-volume circulation has been associated with both normotensive and pre-eclamptic FGR, implicating inadequate plasma volume expansion and uterine artery remodeling as shared pathways (27). These mechanisms provide biological plausibility for PCR, and improving CRF before conception may optimize the maternal hemodynamic starting point, enhance vascular reserve, and improve placental perfusion during the critical first-trimester window when spiral artery remodeling occurs.

The intervention gap: Despite coherent mechanistic data, no intervention trial has tested whether improving preconception cardiovascular function through structured exercise training or a CR-based multicomponent intervention reduces FGR incidence. This is one of the highest priority gaps identified by the narrative evidence map, alongside the absence of any integrated PCR trial. FGR carries both perinatal morbidity and mortality risk and long-term offspring cardiovascular and metabolic risk, so the gap also carries intergenerational consequences.

### 3.5 Narrative evidence map

**Figure 1:**
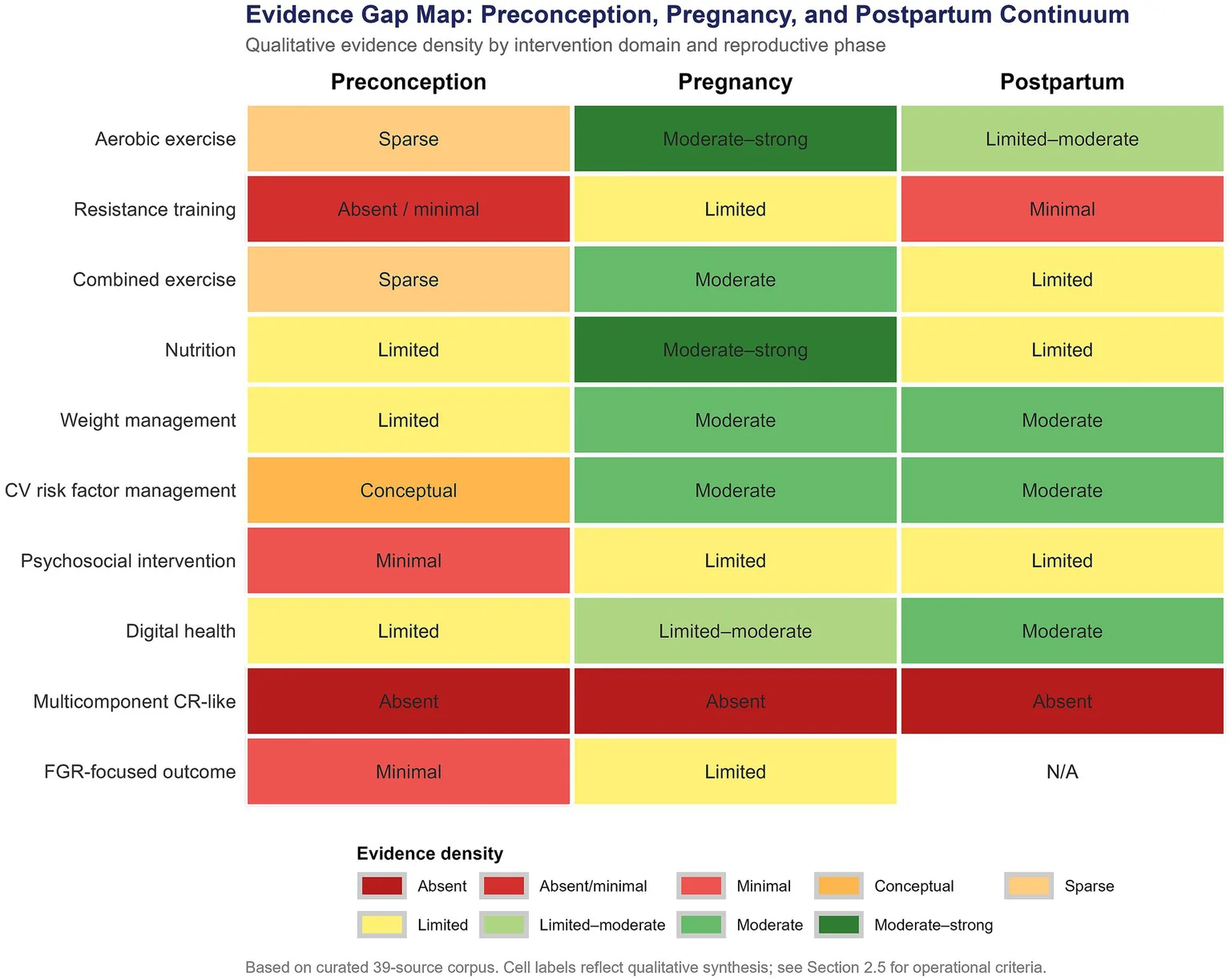
Narrative evidence map across the preconception, pregnancy, and postpartum continuum. Rows represent intervention domains, and columns represent reproductive phases (preconception, pregnancy, and postpartum). Cell labels summarize the density, directness, and maturity of the curated corpus and are not formal certainty ratings. The figure is intended to show evidence distribution and gaps, not pooled effect estimates or guideline recommendations.

Figure 1 and Table 1 summarize the narrative evidence map. Cells describe the relative density and maturity of evidence within the curated corpus, using prespecified qualitative labels from absent to moderate-to-strong. These labels are descriptive and should not be interpreted as formal certainty ratings. Three cross-cutting patterns were apparent.

**Table 1:** Narrative evidence map summary by reproductive phase and intervention type.

Intervention domain	Preconception evidence	Pregnancy evidence	Postpartum evidence	Main gap
Aerobic exercise	Sparse	Moderate to strong	Limited to moderate	Few preconception trials with clinical endpoints
Resistance training	Absent or minimal	Limited	Minimal	Safety and efficacy before conception unknown
Combined exercise	Sparse	Moderate	Limited	No PCR-style protocol
Nutrition	Limited	Moderate to strong	Limited	Few preconception trials powered for APOs
Weight management	Limited	Moderate	Moderate	Optimal timing and dose unclear
CV risk factor management	Conceptual	Moderate	Moderate	Lack of preconception target-driven trials
Psychosocial intervention	Minimal	Limited	Limited	Adherence and acceptability under-studied
Digital health	Limited	Limited to moderate	Moderate	Digital-only effects appear mixed
Multicomponent CR-like model	Absent	Absent	Absent	No integrated PCR trial
FGR-focused outcome	Minimal	Limited	Not applicable	No intervention trial targeting FGR

Pattern 1—Phase asymmetry: The preconception phase is the least studied window across intervention categories. Aerobic exercise, nutrition, and weight management have limited preconception evidence. Resistance training, psychosocial intervention, and integrated multicomponent programs have minimal or absent evidence. This asymmetry may reflect research infrastructure, funding priorities, and the logistical difficulty of recruiting women before pregnancy, rather than biological irrelevance.

Pattern 2—Component isolation. Existing research evaluates individual intervention components (exercise, diet, and weight management) in isolation. No study has tested a multicomponent model analogous to CR, in which patient assessment, exercise prescription, nutrition, risk factor management, psychosocial care, and quality monitoring are delivered as an integrated program. The CR literature demonstrates that multicomponent delivery produces outcomes that isolated components do not achieve, partly through synergistic effects and partly through sustained engagement. Whether similar synergy operates in preconception care is unknown but plausible.

Pattern 3—FGR as the neglected endpoint: Among maternal and fetal outcomes, FGR is the least studied endpoint in preconception intervention research. Most preconception and pregnancy intervention trials use GDM, gestational weight gain, or birth weight as primary outcomes. FGR, which requires serial ultrasound and standardized growth curves for ascertainment, is logistically more demanding to study. The mechanistic data reviewed in Section 3.4 suggest that FGR could be treated as a priority endpoint in future PCR trials.

### 3.6 Evidence quality and heterogeneity

The quality of included evidence was generally high at the aggregate level — approximately two-thirds of sources were Level I — but important heterogeneity limits synthesis. Physical activity measurement ranged from device-based MVPA to single-item self-report. CRF measurement varied from directly measured VO_2max_ to estimated fitness from submaximal protocols. Intervention designs, when present, differed in modality, frequency, intensity, duration, delivery mode, and comparator. Outcome definitions for HDP, GDM, and FGR were not standardized across studies. This heterogeneity precludes formal meta-analysis of preconception-specific effects and reinforces the need for standardized outcome sets in future PCR trials, ideally aligned with the Core Outcome Set for cardiac rehabilitation and the WHO maternal and perinatal health outcome domains.

## 4 Proposed PCR conceptual framework

### 4.1 Eligibility and risk stratification for PCR

PCR is not proposed for all people planning pregnancy. It is most plausibly relevant to women or pregnancy-capable individuals with elevated cardiovascular or obstetric risk, or to those with a history suggesting reduced cardiovascular reserve. A practical eligibility framework could use three tiers.

Tier 1—Established or high-risk CVD: Candidate populations include congenital heart disease, cardiomyopathy, valvular disease, arrhythmia, pulmonary hypertension, prior peripartum cardiomyopathy, or rheumatic heart disease, such as women classified as mWHO II-III or higher where pregnancy is not contraindicated. Referral would usually come from cardiology, cardio-obstetrics, maternal–fetal medicine, or primary care. The proposed intensity is specialist-led PCR integrated with pregnancy heart team review, with exercise prescription only after individualized risk assessment.

Tier 2—Cardiometabolic or prior APO risk: Candidate populations include chronic hypertension, diabetes, obesity, dyslipidemia, prior HDP, preeclampsia, GDM, FGR/SGA, preterm birth, or strong family history of premature CVD. Referral could come from primary care, obstetrics, fertility clinics, endocrinology, or hypertension clinics. The proposed intensity is hybrid CR-informed lifestyle and risk-factor optimization with escalation to cardiology when abnormal testing or symptoms are present.

Tier 3—Lower-risk optimization: Candidate populations include women planning pregnancy with modifiable cardiovascular risk factors but no known CVD. Referral could come from primary care, community health, or digital preconception programs. The proposed intensity is low-intensity education, physical activity counseling, nutrition support, and self-monitoring rather than specialist CR.

Women with pregnancy contraindications under guideline-based risk assessment, unstable symptoms, severe pulmonary hypertension, severe ventricular dysfunction, or other high-risk conditions would require specialist counseling before any exercise-based intervention. For congenital heart disease and rheumatic heart disease, eligibility would need to account for lesion complexity, valve severity, ventricular function, arrhythmia history, anticoagulation, oxygen saturation, pulmonary pressures, prior surgery, and pregnancy risk classification. In these groups, PCR should be understood as a supervised preconception pathway, not a generic exercise program.

**Figure 2:**
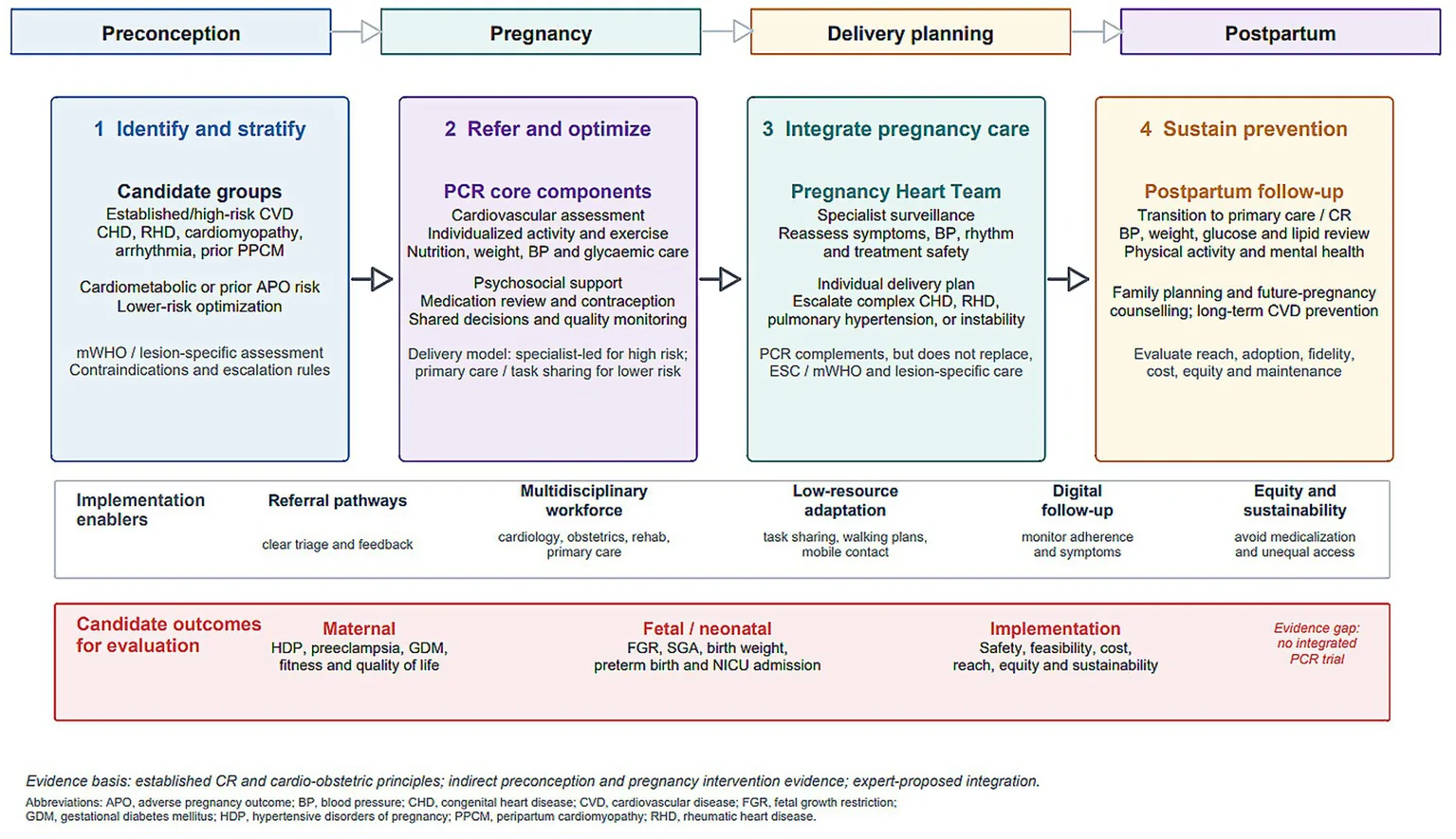
Proposed preconception cardiac rehabilitation pathway across the reproductive continuum. PCR is presented as an expert-proposed adaptation of established cardiac rehabilitation, cardio-obstetric, and implementation-science principles. The pathway begins with eligibility and risk stratification, proceeds through referral triage and multicomponent preconception intervention, and links to pregnancy heart team care and postpartum cardiovascular prevention. Candidate outcomes and implementation metrics require prospective evaluation; the figure does not represent a validated clinical guideline.

Figure 2 summarizes PCR as a provisional adaptation of the 2024 AHA/AACVPR cardiac rehabilitation model. The framework is intended for hypothesis generation and prospective evaluation, not immediate clinical implementation. It may be most relevant to people planning pregnancy who have cardiovascular risk factors, prior adverse pregnancy outcomes, known cardiovascular disease, or assisted reproductive technology exposure (1, 3, 8, 28).

PCR is conceived as assessment-driven, multicomponent, and quality-monitored. Its proposed components are designed to work as a coordinated program, while intervention intensity and care setting would be tailored to cardiovascular risk, functional capacity, reproductive history, and patient preferences.

The framework was derived deductively from established CR components rather than generated inductively from direct PCR trials. Evidence for each component therefore ranges from established CR or guideline support to indirect physiological evidence and expert-proposed adaptation. The component descriptions below retain this distinction explicitly.

### 4.2 Patient assessment preconception cardiovascular readiness assessment

Assessment would begin with referral triage, reproductive intention, current pregnancy status, medication review, and red-flag symptom screening. The responsible lead discipline would vary by risk tier cardiology or cardio-obstetrics for established CVD, maternal–fetal medicine for complex obstetric histories, and primary care or preventive cardiology for lower-risk cardiometabolic optimization.

In standard CR, patient assessment includes cardiovascular history, functional capacity assessment, exercise testing, and risk stratification (8). In PCR, this becomes a preconception cardiovascular readiness assessment with reproductive-specific adaptations.

Core elements: The assessment could include comprehensive cardiovascular and pregnancy history, including prior APOs, mode of delivery, and interpregnancy interval; mWHO, CARPREG II, or ZAHARA scoring when structural or ischemic cardiac disease is present; Life’s Essential eight-assessment covering diet, physical activity, nicotine exposure, sleep health, BMI, blood pressure, blood lipids, and blood glucose; resting blood pressure, lipids, HbA1c, renal function, and selected inflammatory or metabolic markers where indicated; echocardiography when structural heart disease is suspected or known; and body composition assessment beyond BMI, including waist circumference and, where feasible, fat mass quantification (1, 3, 8, 10, 15).

Functional assessment: CPET is the reference standard for functional capacity assessment in CR and, where feasible, could be used in PCR for women with intermediate or high cardiovascular risk. Submaximal testing protocols, field walking tests, or estimated VO_2max_ from validated non-exercise algorithms provide alternatives when CPET is unavailable. The CONCEIVE Study suggests that preconception CPET may reveal hemodynamic phenotypes that are not apparent from resting measurements alone, including low cardiac output, high systemic vascular resistance, and a blunted stroke volume response to exercise (23). Whether exercise training can modify these phenotypes or alter pregnancy outcomes remains unknown.

Risk stratification: Assessment findings could be synthesized into a preconception cardiovascular risk profile that guides intervention intensity, monitoring frequency, and care coordination. Women classified as mWHO Class II–III or with prior APOs, multiple cardiovascular risk factors, or known cardiac disease may warrant coordinated cardiology and maternal-fetal medicine input before and during pregnancy (3, 9, 10). Women with lower risk may be managed predominantly in primary care or community PCR settings. This stratified approach mirrors the risk-stratified delivery of standard CR and is consistent with the cardio-obstetric team-based care model (11, 29).

Evidence basis—Indirect evidence and expert proposal: CR patient assessment components are evidence-based (2024 AHA/AACVPR), and adaptation to the preconception context is expert-proposed. The CONCEIVE Study provides preliminary physiological rationale for preconception CPET as a risk-stratification tool, but its predictive value for pregnancy outcomes has not been validated in intervention trials.

### 4.3 Nutritional counseling preconception cardiometabolic nutrition

PCR nutritional counseling integrates cardiovascular dietary patterns with reproductive nutritional requirements.

Dietary patterns: Mediterranean-style and DASH-style dietary patterns have the strongest evidence base for cardiovascular risk reduction and have been associated with lower risks of preeclampsia, GDM, and preterm birth in observational studies (18). PCR could prioritize these patterns while adapting them to individual preferences, cultural context, and food availability. Core elements include emphasis on vegetables, fruits, whole grains, legumes, nuts, and unsaturated fats; moderation of sodium, added sugars, refined carbohydrates, and saturated fats; and individualized energy balance to support weight management goals.

Reproductive-specific nutrition: Cardiovascular dietary counseling could be integrated with standard preconception nutritional recommendations folic acid supplementation (400–800 μg/day) for neural tube defect prevention, iron status optimization, particularly in women with heavy menstrual bleeding, short interpregnancy intervals, or vegetarian diets, omega-3 fatty acid intake from fish or supplementation, with attention to the inconsistent evidence for omega-3 supplementation and preterm birth prevention, adequate vitamin D, calcium, and iodine, and alcohol cessation before conception. The evidence for most individual micronutrient supplements beyond folic acid in improving pregnancy outcomes is limited, and PCR nutritional counseling could prioritize dietary pattern quality over supplementation.

Weight management integration. Nutritional counseling and weight management (Section 4.3) are operationally linked. In women with obesity, modest preconception weight reduction — 5–10% of body weight — may improve cardiometabolic profile, ovulatory function, and fertility even when ideal BMI is not achieved.

Evidence basis—Indirect evidence: Mediterranean and DASH dietary patterns have cardiovascular outcome evidence in general populations. Prenatal nutrition recommendations (folic acid and iron) are guideline-based. Preconception-specific dietary intervention RCTs with pregnancy endpoints are sparse, and most evidence linking dietary patterns to APO risk is observational.

### 4.4 Weight management and body composition preconception body composition optimization

BMI is an insufficient stand-alone metric for preconception cardiovascular risk assessment and intervention guidance.

Beyond BMI: The dose–response relationship between prepregnancy BMI and HDP is well-established (19), and population-attributable fraction estimates suggest that overweight and obesity account for approximately one quarter of pregnancy complications (20). However, BMI does not distinguish between fat mass and lean mass, does not capture fat distribution, and does not reflect cardiorespiratory fitness — all of which independently modify cardiovascular risk. PCR could incorporate waist circumference, body composition assessment where feasible (bioelectrical impedance analysis or, in specialized settings, dual-energy X-ray absorptiometry), and metabolic markers (fasting glucose, HbA1c, lipid profile, and, where indicated, markers of insulin resistance).

Weight targets: Realistic preconception weight targets could be set collaboratively with the patient, recognizing that weight loss is not a prerequisite for pregnancy in all women with obesity, modest weight reduction (5–10%) may confer clinically meaningful cardiometabolic benefit, the preconception window may be short, particularly in older women or those with diminished ovarian reserve, and aggressive caloric restriction is contraindicated before and during pregnancy. For women with severe obesity (BMI ≥ 40 kg/m^2^) or obesity with metabolic complications, referral for multidisciplinary weight management including medical and, where appropriate, bariatric pathways could be considered — with the important caveat that bariatric surgery requires careful nutritional monitoring during subsequent pregnancy.

Evidence basis: A 2026 systematic review and meta-analysis of preconception lifestyle interventions found that more intensive interventions, particularly those with face-to-face contact and higher session numbers, improved weight and metabolic outcomes, but the effect sizes were modest and the quality of evidence was variable (30). Behavior change techniques associated with greater effectiveness included goal setting, self-monitoring, and social support — techniques that are standard components of CR.

Evidence basis—Indirect to moderate evidence. The BMI–HDP dose–response relationship is well-established (meta-analysis of 157 cohort studies, >50 million pregnancies). Preconception weight loss intervention evidence is limited but growing (2026 systematic review). Body composition measures beyond BMI are rationale-supported, and preconception-specific body composition optimization trials are absent.

### 4.5 Cardiovascular risk factor management preconception life’s essential 8 optimization

PCR could organize cardiovascular risk factor management around Life’s Essential 8, the AHA’s composite cardiovascular health metric, which provides a structured, reproducible, and patient-facing framework.

The Life’s Essential 8 framework. The eight domains — diet quality, physical activity, nicotine exposure, sleep health, BMI, blood pressure, blood lipids, and blood glucose — are each scored from 0 to 100, yielding a composite cardiovascular health score (1, 31). Life’s Essential 8 has been associated with pregnancy outcomes including infertility, and the framework is well-suited to preconception use because it captures modifiable behaviors and risk factors rather than fixed disease states (31).

Medication reconciliation. Pregnancy-incompatible medications require systematic review and replacement before conception. This is particularly relevant for women with established CVD, hypertension, diabetes, or dyslipidemia who may be taking ACE inhibitors, angiotensin receptor blockers, statins, or novel glucose-lowering agents. Medication optimization could be coordinated by cardiology and maternal–fetal medicine specialists and completed before pregnancy since the highest-risk period for teratogenicity is the first trimester — often before pregnancy is recognized and before the first obstetric visit (3, 9, 10).

Target-driven management: CR achieves cardiovascular risk reduction through explicit treatment targets (blood pressure, LDL cholesterol, and HbA1c). PCR could adopt the same principle: preconception blood pressure targets could align with current hypertension guidelines for reproductive-age women, lipid targets could be individualized, recognizing that statins are generally discontinued during pregnancy, but preconception lipid optimization may have lasting vascular benefit, and glycemic targets for women with pre-existing diabetes should follow preconception diabetes guidelines emphasizing tight control before conception. This target-driven approach distinguishes PCR from general preconception counseling, which often addresses risk factors descriptively without explicit numeric goals.

Evidence basis—Indirect evidence. The Life’s Essential 8 framework is evidence-based in general cardiovascular populations; its adaptation to preconception care is expert-proposed. Target-driven management principles are extrapolated from CR. Medication reconciliation is guideline-based. No preconception-specific LE8 intervention trial with pregnancy endpoints exists.

### 4.6 Psychosocial management reproductive cardiovascular mental health

Psychological factors are both an intervention target and an adherence determinant in PCR.

Mental health burden in the preconception population. Women of reproductive age have a high prevalence of depression, anxiety, and psychological distress, and these conditions are more common in women with CVD, obesity, infertility, prior pregnancy loss, or prior APOs — the very populations most likely to benefit from PCR. Psychological distress is independently associated with adverse pregnancy outcomes through behavioral pathways (reduced adherence to health behaviors and prenatal care), physiological pathways (altered hypothalamic–pituitary–adrenal axis function, inflammation, autonomic dysregulation), and treatment-related pathways (psychotropic medication effects on pregnancy). PCR could screen for depression, anxiety, and psychosocial barriers at program entry using validated instruments (PHQ-9, GAD-7) and integrate mental health support when indicated.

Pregnancy-specific psychosocial considerations: PCR would need to address psychosocial factors that are specific to or amplified in the preconception context pregnancy-related anxiety, particularly in women with prior APOs, pregnancy loss, or infertility; fear of exercise during pregnancy, which is prevalent and modifiable through education; body image concerns, which may affect willingness to engage in supervised exercise; partner and family support, which predicts adherence to lifestyle interventions; and health literacy and numeracy, which affect understanding of cardiovascular risk information. Partner involvement may improve adherence, but this strategy has not been evaluated in preconception cardiovascular care and could be studied rather than assumed.

Integration with CR psychosocial practice: Standard CR includes psychosocial assessment and intervention as a core component, and the evidence base for psychosocial benefit in CR is substantial (8). PCR could adopt CR’s psychosocial principles — routine screening, integrated care pathways, and linkage to mental health services — while adapting content to the reproductive context.

Evidence basis—Expert proposal with indirect support. CR psychosocial components are evidence-based. Preconception-specific psychosocial intervention evidence is minimal. The mental health burden in reproductive-age women is well-documented, but intervention trials linking structured psychosocial care to pregnancy outcomes are lacking.

### 4.7 Aerobic exercise training preconception FITT-VP prescription

Aerobic exercise is the most directly transferable component from CR to PCR and the component with the strongest indirect evidence base.

Physiological rationale. Aerobic exercise training improves VO_2max_, cardiac output, stroke volume, endothelial function, blood pressure, insulin sensitivity, and body composition — the same physiological domains implicated in the pathophysiology of HDP, GDM, and possibly FGR. The CONCEIVE study findings that women with low preconception cardiac output and high systemic vascular resistance were at increased risk of PE and FGR provide a specific physiological target for exercise intervention (23).

Proposed FITT-VP prescription. Based on the 2024 AHA/AACVPR CR exercise prescription framework and adapted to the preconception population, an initial PCR exercise prescription includes the following: frequency, three to five sessions per week; intensity, moderate intensity initially (40–59% heart rate reserve, rating of perceived exertion 12–13 on the Borg 6–20 scale), progressing to moderate-to-vigorous as tolerated and indicated; time, 20–60 min per session, progressing from the lower end of this range; type, pregnancy-continuable modalities (walking, cycling, swimming, elliptical training, and low-impact aerobics); volume, target ≥150 min of moderate-intensity aerobic activity per week, consistent with general population and pregnancy physical activity guidelines; and progression, individualized and, where feasible, guided by CPET-derived heart rate zones or heart rate reserve (8, 32).

Safety considerations. Exercise prescription in PCR would need to account for absolute and relative contraindications to exercise, adapted from standard pre-exercise screening tools and modified for the preconception context, the possibility of undetected early pregnancy during the preconception intervention period, which makes pregnancy-continuable modalities and moderate intensity the default, and individual risk profiles, with higher-risk women (mWHO Class II–IV, known coronary artery disease, arrhythmogenic conditions) exercising in supervised settings with appropriate monitoring. The available evidence does not suggest that moderate-intensity exercise harms fertility or early pregnancy in women without contraindications, but this evidence comes from general physical activity studies rather than structured exercise training trials in high-risk preconception populations.

Evidence gap. Although the physiological rationale is strong, no RCT has tested whether structured preconception exercise training — as opposed to general physical activity promotion — improves pregnancy outcomes. The FITT-VP proposal is a starting point for evaluation, not an evidence-based guideline.

Evidence basis—Strongest indirect evidence among all PCR components: FITT-VP prescription is evidence-based in CR (2024 AHA/AACVPR). Preconception physical activity is associated with lower APO risk in observational studies. Physiological rationale is strong (VO_2max_, endothelial function, and insulin sensitivity). No preconception exercise RCT with clinical pregnancy endpoints exists, and the inferential chain relies on physiological rationale and observational associations, not direct intervention evidence.

### 4.8 Strength training preconception resistance and functional training

Resistance training remains a major empty cell in the narrative evidence map and a priority for feasibility research.

Rationale for inclusion: Resistance training improves muscular strength, lean body mass, insulin sensitivity, and functional capacity — outcomes relevant to pregnancy, during which additional body mass would need to be carried and core and pelvic floor strength may influence labor and postpartum recovery. In general CR, resistance training is a core component recommended at 2–3 sessions per week (8). The physiological case for including resistance training in PCR is reasonable, but the evidence to support specific recommendations is absent.

Proposed prescription (starting point for evaluation): Pending dedicated evidence, a conservative starting prescription includes two to three weekly sessions on non-consecutive days, moderate intensity (50–70% estimated one-repetition maximum, or a load permitting 12–15 repetitions with good form before fatigue), major muscle groups, with emphasis on posterior chain, core stability, and pelvic floor musculature, and avoidance of supine exercise in the second and third trimesters if pregnancy occurs during the program.

Safety, feasibility, and evidence gaps: Resistance training during pregnancy is generally considered safe in uncomplicated pregnancies, but preconception resistance training for cardiovascular benefit has not been studied. Key unknowns include adherence in the preconception population, whether women with cardiac disease (mWHO Class II–IV) should avoid heavy resistance training, and whether preconception resistance training influences pregnancy outcomes. Until feasibility and safety data are available, resistance training in PCR could be conservative, supervised, and treated as an adjunct to aerobic training, not a replacement.

Evidence basis—Expert proposal: No preconception-specific resistance training evidence exists. CR resistance training (two to three sessions/week) is evidence-based. Pregnancy resistance training is generally safe in uncomplicated pregnancies, but preconception resistance training for cardiovascular benefit has not been studied. All preconception parameters (frequency, intensity, and modality) are proposed for feasibility evaluation.

### 4.9 Physical activity counseling sustainable behavior change

PCR could not rely solely on supervised exercise sessions. Sustained behavior change requires a counseling framework that addresses motivation, self-efficacy, barriers, and relapse prevention.

Behavior change techniques: Evidence from preconception lifestyle intervention reviews suggests that higher-contact, face-to-face interventions with behavior change techniques — goal setting, self-monitoring, action planning, problem solving, social support, and relapse prevention — are more effective than low-intensity or digital-only programs (30, 33). These techniques are established components of CR and are directly transferable to PCR.

Digital and wearable technology. Wearable devices (accelerometers, heart rate monitors) and smartphone applications can support self-monitoring, provide feedback, and enable remote program delivery. However, the evidence for stand-alone digital interventions in preconception care is mixed. A 2025 systematic review of web-based preconception interventions found that digital-only programs had small and inconsistent effects on clinical outcomes, with engagement declining over time (33). Digital tools are best understood as adjuncts to, not replacements for, structured in-person or hybrid PCR delivery. Hybrid models—combining initial in-person assessment and supervised sessions with digital self-monitoring and periodic remote coaching—are plausible but unevaluated in preconception cardiovascular care.

Adherence and engagement: Adherence is the Achilles’ heel of lifestyle interventions. CR completion rates vary widely across programs and populations, and preconception interventions face the additional challenge that participants are not responding to a clinical event (as in standard CR) but preparing for a future event. This “prehabilitation” framing—exercise and lifestyle change to prepare for a future stressor rather than to recover from a past one—may require different motivational strategies. Research on prehabilitation adherence in surgical populations may provide a model, but preconception-specific adherence research is needed.

Evidence basis—Indirect evidence: Behavior change techniques (goal setting, self-monitoring, and action planning) are evidence-based in general populations and CR. Preconception-specific counseling evidence is limited; higher-contact face-to-face interventions appear more effective than digital-only programs, but the evidence is from a small number of heterogeneous studies. Digital adjuncts show mixed results.

### 4.10 Program quality PCR quality metrics

Program quality measurement is what distinguishes PCR from general preconception health advice. CR has a well-established tradition of program certification, audit, and quality improvement, and PCR could adopt the same principle from inception.

Proposed quality domains and candidate metrics.

Referral and enrollment proportion of eligible women referred to PCR who enroll.

Program completion proportion of enrolled women completing the prescribed PCR program.

Intervention fidelity adherence to prescribed exercise frequency, intensity, and duration, attendance at nutrition and psychosocial sessions.

Cardiovascular health improvement: change in Life’s Essential 8 composite score from program entry to completion.

Functional capacity change in VO_2_max, 6-min walk distance, or equivalent from program entry to completion.

Risk factor control change in blood pressure, lipids, HbA1c, and weight from program entry to completion.

Pregnancy outcomes (where applicable): APO incidence, including HDP, preeclampsia, GDM, preterm birth, and FGR or SGA, mode of delivery, and birthweight.

Postpartum follow-up proportion of women completing postpartum cardiovascular risk reassessment, consistent with the 2024 AHA postpartum scientific statement (34).

Patient-reported outcomes: quality of life, exercise self-efficacy, program satisfaction, and perceived cardiovascular health competence.

Quality measurement as a research tool. In the early phase of PCR development, quality measurement serves dual purposes: program improvement and research. Standardized quality metrics could enable comparison across PCR delivery models, populations, and settings, identify components associated with the largest cardiovascular health improvements, and provide the outcome data needed for definitive RCTs. Without quality measurement, PCR would be difficult to evaluate as a reproducible intervention.

Evidence basis—Expert proposal. CR quality measurement and program certification are evidence-based practices with established audit frameworks. All PCR quality metrics are proposed for evaluation, and no data exist on PCR program performance. The quality framework is adapted from CR audit principles and could be validated alongside PCR feasibility trials (Table 2).

**Table 2:** Proposed adaptation of cardiac rehabilitation components to PCR.

CR component	PCR adaptation	Candidate measures
Patient assessment	Preconception cardiovascular readiness assessment	mWHO, CARPREG II, Life’s Essential 8, CPET, echocardiography
Nutritional counseling	Cardiometabolic and reproductive nutrition	DASH/Mediterranean diet adherence, micronutrient status
Weight/body composition	Preconception body composition optimization	BMI, waist circumference, body composition, metabolic markers
Risk factor management	Life’s Essential 8 target optimization	BP, lipids, HbA1c, smoking, sleep, activity
Psychosocial care	Reproductive cardiovascular mental health	PHQ-9, GAD-7, social support, adherence barriers
Aerobic exercise	Preconception FITT-VP prescription	VO_2max_, HRR, weekly MVPA, session adherence
Strength training	Resistance and functional preparation	1-RM or estimated RM, pelvic floor/core function
Physical activity counseling	Sustainable behavior change	Wearable data, self-monitoring, sedentary time
Program quality	PCR implementation metrics	Enrollment, completion, CVH improvement, APOs, FGR/SGA

### 4.11 Component integration and sequencing

Referral pathway and follow-up. A feasible pathway could begin when a woman planning pregnancy is identified in primary care, cardiology, obstetrics, fertility care, or postpartum follow-up after an APO. Initial triage would assign a risk tier and determine whether pregnancy heart team review is required before exercise prescription. The active PCR phase would include individualized goals, exercise and nutrition prescriptions, risk-factor optimization, and psychosocial screening. Follow-up checkpoints could occur at program entry, 6–12 weeks, readiness for conception, early pregnancy booking, mid-pregnancy cardiovascular review when indicated, 6–12 weeks postpartum, and 6–12 months postpartum.

Responsible disciplines. PCR would require a scalable team rather than a single specialty. Core roles include cardiology or preventive cardiology for cardiovascular risk and exercise safety, maternal–fetal medicine or obstetrics for reproductive risk, rehabilitation professionals or exercise physiologists for FITT–VP prescription, dietitians for nutrition and weight management, nurses or midwives for care coordination, psychologists or counselors for psychosocial support, and primary care for long-term follow-up.

Sustainability in health systems. In settings with established CR and cardio-obstetric services, PCR could be embedded as a preconception referral stream within existing CR programs. In settings where both services are limited, a hub-and-spoke model may be more realistic: specialist hubs define protocols and supervise high-risk cases, while community clinics deliver low-risk counseling, blood pressure and glucose monitoring, walking-based exercise plans, and digital follow-up.

The proposed components are intended to interact, although their optimal sequence and combined effects remain unknown.

Component interactions. Aerobic exercise enhances insulin sensitivity, which amplifies the effect of nutritional counseling on glycemic control. Nutritional counseling supports exercise adaptation and body composition change. Psychosocial management addresses barriers to exercise and nutrition adherence. Weight management success depends on integrated exercise, nutrition, and behavior change support. Cardiovascular risk factor management is the quantitative target that exercise, nutrition, and weight management collectively serve. These interactions are the rationale for multicomponent delivery; a program that delivers exercise without nutrition support, or nutrition counseling without psychosocial screening, underperforms relative to integrated delivery.

Provisional sequencing for feasibility studies. A PCR program could be evaluated in three phases: assessment and planning, active multicomponent intervention, and transition to maintenance and pregnancy care. The illustrative durations of 1–4, 5–16, and 17–24 + weeks are starting points for feasibility testing, not validated treatment periods, and would require individualization.

Integration with cardio-obstetric care. PCR extends cardio-obstetric care backward into the preconception period. Women completing PCR could transition seamlessly to pregnancy heart team care when pregnancy occurs, with shared health records, consistent risk stratification, and coordinated postpartum follow-up. This closes the loop from preconception cardiovascular optimization through pregnancy management to postpartum cardiovascular prevention (11, 34).

Alignment with ESC guidance. The 2025 ESC Guidelines for the management of cardiovascular disease and pregnancy emphasize pre-pregnancy counseling, multidisciplinary Pregnancy Heart Team care, individualized risk stratification, and follow-up across pregnancy and the postpartum period (35). PCR is proposed to sit upstream of this pathway. It would not replace ESC risk assessment, mWHO classification, lesion-specific counseling, or contraindication-based advice. Instead, PCR could provide a structured method for implementing modifiable cardiovascular optimization before pregnancy in women whose guideline-based assessment supports pregnancy planning.

## 5 Implementation considerations and risks

### 5.1 Implementation in low-resource settings and LMICs

The largest implementation challenge is that conventional CR and specialized cardio-obstetric services are absent or inaccessible in many regions. A PCR model that depends on tertiary CR infrastructure would therefore risk widening inequity. For LMICs and under-resourced settings, the framework would need adaptation around low-cost risk stratification, task sharing, and referral thresholds rather than comprehensive hospital-based rehabilitation.

A pragmatic LMIC pathway could include community blood pressure measurement, diabetes and anemia screening, body mass index and waist assessment, symptom-based cardiovascular triage, simple functional capacity measures, such as the 6-min walk test, walking-based exercise prescriptions, group education, and mobile-phone follow-up. High-risk findings would trigger referral to regional cardiology or obstetric medicine services. This approach would need local validation because safety thresholds, referral capacity, cultural acceptability, and costs differ substantially across health systems.

Implementation-science evaluation could use Proctor et al.’s outcomes of acceptability, adoption, appropriateness, feasibility, fidelity, cost, penetration, and sustainability (36). RE-AIM could be used to evaluate reach and maintenance (37), whereas CFIR could help identify referral, staffing, adherence, financing, and health-system determinants that shape implementation (38). Together, these frameworks could distinguish whether a future PCR program me underperforms because of its underlying intervention or because of delivery failure.

### 5.2 Potential disadvantages, costs, and risks

PCR may have disadvantages. It could medicalize preconception care, increase anxiety in women planning pregnancy, divert limited CR capacity away from established cardiac patients, and create inequitable access if offered mainly to women with private insurance or tertiary-center care. Exercise testing and supervised training also carry costs and may be unnecessary for lower-risk women who can be supported through primary care and community physical activity counseling.

Clinical risks must also be considered. In women with complex congenital heart disease, rheumatic valvular disease, pulmonary hypertension, arrhythmias, ventricular dysfunction, or anticoagulation needs, generic exercise advice could be unsafe. PCR would therefore require contraindication screening, escalation rules, and individualized prescriptions. These concerns support a tiered model in which high-risk women receive specialist-supervised care and lower-risk women receive proportionate, lower-intensity support.

A counterargument is that the current evidence does not justify building a new program before efficacy is known. We partly agree. The framework should not be promoted as standard care until feasibility, safety, implementation, and outcome studies are available. Its immediate value is to specify a testable pathway and to make the assumptions behind preconception cardiovascular optimization explicit.

A likely critique is that CR is a secondary prevention model for people with established CVD, whereas many women planning pregnancy are not cardiac patients. The concern deserves a direct response.

PCR differs from conventional post-event CR in timing, population, and reproductive safety considerations. Its closer analog is prehabilitation: preparation for a predictable cardiovascular stressor rather than recovery after an event.

PCR is therefore proposed as cardiovascular prehabilitation for pregnancy, pending consensus, feasibility, and efficacy testing.

## 6 Research agenda

### 6.1 Priority 1: framework development and efficacy testing

An international Delphi process could define candidate components, eligibility criteria, contraindications, outcome metrics, and minimum intervention dose. Subsequent multicenter trials could compare PCR with usual preconception care and evaluate maternal, fetal, neonatal, and cardiovascular outcomes.

### 6.2 Priority 2: mechanistic and feasibility studies

Prospective cohort studies could examine whether preconception CPET metrics predict pregnancy hemodynamics, placental biomarkers, and APOs. Feasibility trials should evaluate PCR adherence, safety, acceptability, and improvement in Life’s Essential 8 metrics. Mechanistic studies could assess whether preconception cardiorespiratory fitness relates to placental angiogenic markers, uterine artery Doppler indices, and fetal growth.

### 6.3 Priority 3: implementation and equity studies

Implementation studies could compare hybrid and face-to-face PCR delivery, assess cost-effectiveness, and evaluate women’s preferences, adherence barriers, referral completion, workforce needs, and sustainability. LMIC-adapted PCR models could be developed using low-cost functional testing, task sharing, community delivery platforms, and clear escalation thresholds. Implementation outcomes should be reported alongside clinical outcomes so that feasibility, fidelity, reach, adoption, equity, and maintenance can be evaluated explicitly (36–38).

## 7 Limitations

This review has several limitations. Source selection was purposive rather than exhaustive, screening was not duplicated, total database yield was not retained as a reliable denominator, and formal risk-of-bias appraisal was not performed. No PRISMA flow diagram is provided because the review was not conducted as a systematic or scoping review. The narrative evidence map may therefore omit relevant studies and should not be interpreted as a formal systematic map. The PCR framework extrapolates from CR, cardio-obstetrics, implementation science, and indirect physiological evidence because integrated PCR trials do not exist. Evidence is concentrated in high-income settings, LMIC-specific implementation evidence is limited, and congenital heart disease and rheumatic heart disease populations require lesion-specific eligibility and safety criteria.

## 8 Conclusion

This structured narrative review identifies a substantial preconception evidence gap and proposes PCR as a testable cardiovascular prehabilitation framework. Current evidence supports its biological rationale but not its clinical efficacy. The framework is therefore best viewed as a research and implementation agenda that specifies candidate populations, referral routes, responsible disciplines, follow-up points, and potential barriers. Consensus development, feasibility evaluation, implementation-science studies, cost analyses, and randomized testing are required before PCR can be recommended as a care model.

## Glossary

AHA: American Heart Association
APO: Adverse pregnancy outcome
BMI: Body mass index
CARPREG II: Cardiac Disease in Pregnancy Study II risk score
CNKI: China National Knowledge Infrastructure
CPET: Cardiopulmonary exercise testing
CR: Cardiac rehabilitation
CVD: Cardiovascular disease
CVH: Cardiovascular health
FGR: Fetal growth restriction
FITT-VP: Frequency, intensity, time, type, volume, progression
GDM: Gestational diabetes mellitus
HDP: Hypertensive disorders of pregnancy
LMIC: Low- and middle-income country
mWHO: Modified World Health Organization maternal cardiovascular risk classification
PCR: Preconception cardiac rehabilitation
PRISMA-ScR: Preferred Reporting Items for Systematic Reviews and Meta-Analyses Extension for Scoping Reviews
RCT: Randomized controlled trial
SGA: Small for gestational age
ZAHARA: Zwangerschap bij Aangeboren HARtAfwijkingen risk score
