# Supplementary material for: Preconception cardiac rehabilitation: a structured narrative review and conceptual framework for maternal cardiovascular health and fetal growth
Source: Front Med (Lausanne). 2026 Aug 13;13:1914342. doi: 10.3389/fmed.2026.1914342 (PMC13518555; doi:10.3389/fmed.2026.1914342)
Supplement: Supplementary file 1 [file Supplementary_file_1.docx]

**Supplementary Material**

**Preconception Cardiac Rehabilitation: A Structured Narrative Review and Conceptual Framework for Maternal Cardiovascular Health and Fetal Growth**

**Supplementary File 1. Complete Search Strategies**

**Search 1: Core Search (Preconception x Exercise/CR x Pregnancy Outcomes)**

(("Preconception Care"[Mesh] OR preconception[tiab] OR "pre-pregnancy"[tiab]

OR prepregnancy[tiab] OR "before pregnancy"[tiab] OR "preconceptional"[tiab])

AND

("Cardiac Rehabilitation"[Mesh] OR "Exercise Therapy"[Mesh] OR "Exercise"[Mesh]

OR "Physical Fitness"[Mesh] OR "cardiac rehabilitation"[tiab]

OR "cardiovascular rehabilitation"[tiab] OR "exercise training"[tiab]

OR "physical activity"[tiab] OR "aerobic exercise"[tiab]

OR "cardiorespiratory fitness"[tiab] OR "exercise capacity"[tiab]

OR "aerobic fitness"[tiab] OR "lifestyle intervention"[tiab])

AND

("Pregnancy Complications"[Mesh] OR "Hypertension, Pregnancy-Induced"[Mesh]

OR "Pre-Eclampsia"[Mesh] OR "Diabetes, Gestational"[Mesh]

OR "Premature Birth"[Mesh] OR "Fetal Growth Retardation"[Mesh]

OR "adverse pregnancy outcome"[tiab] OR preeclampsia[tiab]

OR "gestational diabetes"[tiab] OR "gestational hypertension"[tiab]

OR "preterm birth"[tiab] OR "fetal growth"[tiab] OR FGR[tiab]

OR IUGR[tiab] OR "small for gestational age"[tiab] OR SGA[tiab]

OR "low birth weight"[tiab]))

**Search 2: Preconception x Cardiovascular Risk Factor Management**

(("Preconception Care"[Mesh] OR preconception[tiab] OR prepregnancy[tiab]

OR "pre-pregnancy"[tiab])

AND

("Cardiovascular Diseases/prevention and control"[Mesh]

OR "Heart Disease Risk Factors"[Mesh] OR "Risk Reduction Behavior"[Mesh]

OR "cardiovascular health"[tiab] OR "cardiovascular risk"[tiab]

OR "blood pressure"[tiab] OR "lipid"[tiab] OR "glucose"[tiab]

OR "weight management"[tiab] OR "body composition"[tiab]

OR "Life's Essential 8"[tiab] OR "cardiovascular optimization"[tiab])

AND

("Pregnancy Outcome"[Mesh] OR "adverse pregnancy outcome"[tiab]

OR preeclampsia[tiab] OR "gestational diabetes"[tiab]

OR "preterm birth"[tiab] OR "maternal morbidity"[tiab]

OR "maternal mortality"[tiab]))

**Search 3: Cardio-Obstetrics x Preconception Care**

("cardio-obstetric"[tiab] OR "cardio obstetric"[tiab]

OR "pregnancy heart team"[tiab] OR "obstetric cardiac"[tiab]

OR "cardiac disease in pregnancy"[tiab] OR "cardiovascular disease in pregnancy"[tiab])

AND

(preconception[tiab] OR prepregnancy[tiab] OR "pre-pregnancy"[tiab]

OR "before pregnancy"[tiab] OR "preconceptional"[tiab]

OR "pregnancy planning"[tiab] OR "reproductive counseling"[tiab])

**Search 4: Cardiac Rehabilitation Core Components Framework**

("Cardiac Rehabilitation"[Mesh] OR "cardiac rehabilitation"[tiab])

AND

("core component"[tiab] OR framework[tiab] OR model[tiab]

OR guideline[tiab] OR "scientific statement"[tiab]

OR "exercise prescription"[tiab] OR "FITT"[tiab]

OR "program quality"[tiab] OR "delivery model"[tiab]

OR "virtual rehabilitation"[tiab] OR "remote rehabilitation"[tiab])

AND

("Guideline"[ptyp] OR "Practice Guideline"[ptyp]

OR "Scientific Statement" OR review[ptyp] OR systematicreview[ptyp])

**Search 5: Postpartum Cardiovascular Outcomes and Cardiac Rehabilitation**

("Postpartum Period"[Mesh] OR postpartum[tiab] OR "post-partum"[tiab]

OR postnatal[tiab] OR "fourth trimester"[tiab] OR "after delivery"[tiab])

AND

("Cardiac Rehabilitation"[Mesh] OR "Exercise Therapy"[Mesh]

OR "cardiac rehabilitation"[tiab] OR "exercise intervention"[tiab]

OR "lifestyle intervention"[tiab] OR "cardiovascular prevention"[tiab]

OR "risk reduction"[tiab] OR "blood pressure management"[tiab])

AND

("adverse pregnancy outcome"[tiab] OR preeclampsia[tiab]

OR "gestational diabetes"[tiab] OR "gestational hypertension"[tiab]

OR "preterm birth"[tiab] OR "cardiovascular risk"[tiab]

OR "cardiovascular disease"[tiab])

**Search 6: Preconception Exercise Physiology x FGR/SGA**

(("Preconception Care"[Mesh] OR preconception[tiab] OR prepregnancy[tiab])

AND

("Physical Fitness"[Mesh] OR "Exercise Test"[Mesh]

OR "cardiorespiratory fitness"[tiab] OR VO2max[tiab] OR VO2peak[tiab]

OR "exercise capacity"[tiab] OR CPET[tiab]

OR "cardiopulmonary exercise"[tiab] OR "hemodynamic"[tiab]

OR "cardiac output"[tiab] OR "stroke volume"[tiab]

OR "endothelial function"[tiab] OR "vascular resistance"[tiab])

AND

("Fetal Growth Retardation"[Mesh] OR "fetal growth"[tiab] OR FGR[tiab]

OR IUGR[tiab] OR "small for gestational age"[tiab] OR SGA[tiab]

OR "placental insufficiency"[tiab] OR "placental function"[tiab]

OR "birth weight"[tiab] OR "birthweight"[tiab]))

**Supplementary File 2. Eligibility, Risk Stratification, and Curated Source Selection Framework**

**Methodological note: This structured narrative review used database searches and expert knowledge to support purposive curation of the evidence base. The narrative evidence map was descriptive and secondary to framework development. It was not a formal systematic evidence gap map, systematic review, or PRISMA-ScR-compliant scoping review.**

**1. Curated Source Selection Flow**

┌─────────────────────────────────────────────────────────────────┐

│	IDENTIFICATION	│

├─────────────────────────────────────────────────────────────────┤

│	│

│ Structured search sources: Expert knowledge sources: │

│ ├─ PubMed (core searches)	├─ Citation tracking	│

│ ├─ Scopus	├─ Society scientific	│

│ ├─ Web of Science	statements	│

│ ├─ Cochrane Library	├─ Reference lists of	│

│ ├─ CNKI	key reviews	│

│ └─ Wanfang	└─ Research team expertise│

│	│	│

│	▼	│

├─────────────────────────────────────────────────────────────────┤

│	CURATION	│

├─────────────────────────────────────────────────────────────────┤

│	│

│ Structured search outputs and expert-identified sources	│

│	│	│

│	├── Four sequential curation questions: │

│	│	1. Is the source relevant to PCR framework development?│

│	│	2. Is the evidence direct, authoritative, or otherwise │

│	│	informative for the stated question? │

│	│	3. Does it address a relevant intervention, phase, or │

│	│	maternal/fetal/postpartum outcome domain? │

│	│	4. Does it add information not already represented? │

│ Full-text retrieval and framework-focused assessment	│

│	│	│

│	├── Priority ranking: │

│	│	1. AHA/ESC/ACOG scientific statements & guidelines │

│	│	2. Systematic reviews & meta-analyses	│

│	│	3. Key RCTs & prospective cohort studies	│

│	│	4. Cardio-obstetrics pathway papers & narrative	│

│	│	reviews	│

│	│	5. Mechanistic studies (maternal CV–placental–fetal │

│	│	growth)	│

│	▼	│

├─────────────────────────────────────────────────────────────────┤

│	INCLUDED	│

├─────────────────────────────────────────────────────────────────┤

│	│

│ Curated source set (n = 39; not a systematic screening yield)	│

│ ├─ AHA/ESC scientific statements (n = 6)	│

│ ├─ Systematic reviews / meta-analyses (n = 14)	│

│ ├─ Cardio-obstetrics reviews / pathway papers (n = 5)	│

│ ├─ Narrative evidence-mapping methodology context (n = 1)	│

│ ├─ Pregnancy / postpartum intervention reviews (n = 6)	│

│ └─ Mechanistic studies (n = 5)	│

│	│

│ Evidence levels describe study design, not a formal certainty rating.│

│ All sources are from indexed, peer-reviewed journals or	│

│	professional society statements. │

│ Source years: 2014-2026; searches prioritized 2020-2026, with	│

│	│

└─────────────────────────────────────────────────────────────────┘

**Methodological boundaries:**

- Sources were purposively curated; duplicate independent screening was not performed.

- Exported records were not retained as one reliable deduplicated denominator; therefore, no total database yield, stage-specific exclusions, or PRISMA flow diagram is reported.

- Selection was guided by relevance, directness, authority, applicability to the PCR framework, and contribution beyond sources already represented.

- These boundaries are reported in the revised manuscript Methods and Limitations sections.

**2. Inclusion and Exclusion Criteria**

**Inclusion Criteria**

| Element | Criterion |
| --- | --- |
| Domain | Preconception cardiovascular health; CR component adaptation for the preconception period; cardio-obstetric preconception care; preconception or pregnancy exercise / diet / lifestyle interventions; postpartum cardiovascular prevention after APOs; mechanistic links between maternal cardiovascular physiology and fetal growth |
| Study design | RCTs, prospective/retrospective cohorts, case-control studies, systematic reviews/meta-analyses, clinical practice guidelines, AHA/ESC scientific statements, narrative reviews (for background and framework construction) |
| Language | English or Chinese |

**Exclusion Criteria**

- Non-human studies

- Case reports

- Non-cardiovascular preconception interventions unrelated to the framework

- Conference abstracts with insufficient data

- Studies unrelated to maternal or fetal outcomes

**3. Evidence Classification and Appraisal Boundaries**

| Level | Study Design | Approximate Source Count |
| --- | --- | --- |
| I | Systematic reviews/meta-analyses, scientific statements, reporting guidelines | Approximately 20+ |
| II | RCTs | Few |
| IV | Cohort studies, case-control studies | Several |
| V | Narrative reviews, protocols, pathway papers | Several |

**Note: Evidence classification reflects study design and is interpreted alongside directness, consistency, and applicability to the proposed PCR framework. Formal risk-of-bias and guideline-appraisal tools (AMSTAR-2, RoB 2, ROBINS-I, and AGREE II) were not applied. Therefore, labels in this supplement do not represent formal certainty ratings.**

**4. Narrative Evidence-Mapping Framework**

**Row Dimension: Intervention Types**

1. Aerobic exercise training

2. Resistance training

3. Combined exercise (aerobic + resistance)

4. Nutritional intervention

5. Weight management / body composition optimization

6. Comprehensive CV risk factor management (BP / glucose / lipids)

7. Psychosocial intervention

8. Multicomponent lifestyle intervention (3 or more CR components)

9. Digital health / remote intervention

10. Health system / policy intervention

**Column Dimension: Outcomes**

1. Hypertensive disorders of pregnancy (HDP)

2. Preeclampsia (PE)

3. Gestational diabetes mellitus (GDM)

4. Preterm birth (PTB)

5. Cesarean delivery rate

6. Postpartum cardiovascular outcomes

7. Fetal growth restriction (FGR)

8. Small for gestational age (SGA)

9. Low birth weight (LBW)

10. NICU admission

11. Offspring cardiovascular remodeling

**Reproductive Phase Stratification**

- Preconception intervention

- Pregnancy intervention

- Postpartum intervention

**Narrative Evidence-Density Labels (qualitative, not certainty ratings)**

| Label | Meaning | Operational Criterion |
| --- | --- | --- |
| Absent | No published intervention studies | Search returned no relevant studies |
| Absent or minimal | Isolated case reports or minimal indirect evidence | 1 source, insufficient to draw conclusions |
| Minimal | Very limited evidence | 1–2 sources, cannot draw conclusions |
| Sparse | Preliminary evidence, not suitable for synthesis | 3–5 sources, high heterogeneity |
| Limited | Some evidence but conclusions uncertain | Multiple studies, directionally consistent but small samples |
| Limited to moderate | Emerging evidence base | Several studies, at least 1 SR/MA |
| Moderate | Substantial synthesizable evidence | Multiple studies, 1 or more SR/MA |
| Moderate to strong | Robust evidence base | Multiple high-quality SR/MA + scientific statements |
| Strong | Well-established conclusions | Multiple independently validated RCTs or large-scale evidence base |

**5. Candidate Eligibility and Risk Stratification for PCR**

Tier 1: established or high-risk cardiovascular disease, including complex congenital heart disease, cardiomyopathy, clinically significant valvular or rheumatic heart disease, arrhythmias, pulmonary hypertension, prior peripartum cardiomyopathy, or mWHO class II-III or higher when pregnancy is not contraindicated. These individuals require specialist-led assessment before exercise prescription.

Tier 2: cardiometabolic risk or previous adverse pregnancy outcomes, including chronic hypertension, diabetes, obesity, dyslipidemia, prior hypertensive disorders of pregnancy, gestational diabetes, preterm birth, fetal growth restriction, or small-for-gestational-age birth. These individuals may be managed through structured primary-care and specialist-supported pathways.

Tier 3: lower-risk people planning pregnancy who seek cardiovascular health optimization. Proportionate lifestyle counselling and community-based support may be appropriate, with escalation when concerning symptoms or screening findings arise.

## 6. Implementation Boundary, Risks, and Equity

PCR is an expert-proposed, testable framework rather than a validated care model. In settings without established cardiac rehabilitation or cardio-obstetric services, an adapted model may rely on task sharing, basic blood pressure, diabetes, anemia, and symptom screening, simple functional assessment, walking-based activity prescriptions, mobile follow-up, and explicit referral thresholds. These adaptations require local safety, acceptability, cost, and equity evaluation.

Potential disadvantages include medicalization of preconception care, anxiety, diversion of limited rehabilitation resources, inequitable access, testing and supervision costs, and inappropriate generic exercise advice for women with complex congenital heart disease, rheumatic valvular disease, pulmonary hypertension, arrhythmias, ventricular dysfunction, or anticoagulation needs. These concerns support tiered referral, contraindication screening, and individualized prescriptions.

7. Data Extraction Template

| Field | Description |
| --- | --- |
| Source ID | First author + year |
| Source type | Scientific statement / systematic review / RCT / cohort / review / mechanism |
| Country/Region | Country or region of the research |
| Study design | Original study design (if applicable) |
| Population | Age / parity / underlying conditions |
| Sample size | Total sample size or number of included studies (for reviews) |
| Intervention type | Classified by CR core components |
| Intervention timing | Preconception / pregnancy / postpartum |
| Key findings | Core findings relevant to the PCR framework |
| Domain relevance | CR core component(s) addressed |
| Evidence level | Level I–V |
| Notes | Limitations, citation chains, relationships to other sources |

**Supplementary File 3. Annotated Bibliography**

**CATEGORY A: CORE FRAMEWORKS (7 sources)**

**[A1] Brown, T. M., Pack, Q. R., Aberegg, E., Brewer, L. C., Ford, Y. R., Forman, D. E., Gathright, E. C., Khadanga, S., Ozemek, C., & Thomas, R. J. (2024).** Core Components of Cardiac Rehabilitation Programs: 2024 Update: A Scientific Statement From the American Heart Association and the American Association of Cardiovascular and Pulmonary Rehabilitation. *Circulation*, 150(18), e328–e347. doi:10.1161/CIR.0000000000001289

*Evidence Level: I (AHA Scientific Statement)*

**Key findings:** Updates the 2007 AHA/AACVPR CR statement with nine core components. New: Program Quality added as a standalone component. First formal recognition of virtual/remote delivery models. FITT-VP framework specified for aerobic (3–5×/wk, 40–89% HRR, 20–60 min) and resistance training (10–15 RM, 2–3×/wk). Health equity explicitly addressed.

**Mapping to PCR:** Primary source for the PCR nine-component adaptation framework.

**Quality: Level I; professional society statement; peer-reviewed Circulation.**

**[A2] Khan, S. S., Brewer, L. C., Canobbio, M. M., Cipolla, M. J., Grobman, W. A., Lewey, J., Michos, E. D., Miller, E. C., Perak, A. M., Wei, G. S., & Gooding, H. (2023).** Optimizing Prepregnancy Cardiovascular Health to Improve Outcomes in Pregnant and Postpartum Individuals and Offspring: A Scientific Statement From the American Heart Association. *Circulation*, 147(7), e76–e91. doi:10.1161/CIR.0000000000001124

*Evidence Level: I (AHA Scientific Statement)*

**Key findings:** Unfavorable cardiovascular health is highly prevalent in US reproductive-age women. Prepregnancy CVH is linked to APOs in a graded fashion. Biological processes leading to APOs begin before conception. Applies a life-course and intergenerational framework to measure, modify, and monitor prepregnancy CVH. Recommends the Life's Essential 8 framework. Calls for clinical trials of prepregnancy interventions.

**Mapping to PCR:** Theoretical foundation justifying the preconception window as critical. LE8 provides measurable targets for PCR cardiovascular risk factor management.

**Quality: Level I; professional society statement; peer-reviewed Circulation.**

**[A5] Milani, J. G. P. O., Milani, M., Verboven, K., Cipriano, G., & Hansen, D. (2024).** Exercise Intensity Prescription in Cardiovascular Rehabilitation: Bridging the Gap Between Best Evidence and Clinical Practice. *Frontiers in Cardiovascular Medicine*, 11, 1380639. doi:10.3389/fcvm.2024.1380639

*Evidence Level: V (Review)*

**Key findings:** FITT-VP framework for CR: Frequency 3–5×/wk; Intensity based on CPET/ventilatory thresholds (VT1/VT2); Time 20–60 min; Type = endurance + resistance; Volume ≥500–1000 MET-min/wk; Progression individualized. When CPET is unavailable: 6-min walk test + Borg RPE + Talk Test as substitutes. Emphasizes aligning workouts with individual physiological changes.

**Mapping to PCR:** Direct source for PCR exercise prescription (FITT-VP adaptation).

**Quality:** Level V; peer-reviewed Front Cardiovasc Med; indexed in WoS/Scopus.

**[A6] Mauricio, R., Sharma, G., Lewey, J., Tompkins, R., Plowden, T., Rexrode, K., Canobbio, M., Skowronski, J., Hameed, A., Silversides, C., Reynolds, H., & Vaught, A. (2025).** Assessing and Addressing Cardiovascular and Obstetric Risks in Patients Undergoing Assisted Reproductive Technology: A Scientific Statement From the American Heart Association. *Circulation*, 151(8). doi:10.1161/CIR.0000000000001292

*Evidence Level: I (AHA Scientific Statement)*

**Key findings:** Reviews the ART process and known short- and long-term risks associated with ART that can adversely affect patients with CVD. Provides a suggested approach to evaluating and counseling patients with CVD contemplating ART and suggested management before and during the ART process.

**Mapping to PCR: Identifies ART users as a relevant subgroup for preconception cardiovascular optimization.**

**Quality:** Level I; AHA; peer-reviewed Circulation.

**[A7] Parikh, N. I., Gonzalez, J. M., Anderson, C. A. M., Judd, S. E., Rexrode, K. M., Hlatky, M. A., Gunderson, E. P., Stuart, J. J., & Vaidya, D. (2021).** Adverse Pregnancy Outcomes and Cardiovascular Disease Risk: Unique Opportunities for Cardiovascular Disease Prevention in Women: A Scientific Statement From the American Heart Association. *Circulation*, 143(18), e902–e916. doi:10.1161/CIR.0000000000000961

*Evidence Level: I (AHA Scientific Statement)*

**Key findings:** APOs (HDP, GDM, PTB, SGA, recurrent pregnancy loss) independently increase long-term CVD risk. Pregnancy serves as a "window to future cardiovascular health." Recommends integrating pregnancy history into CVD risk assessment. Highlights the importance of adopting a heart-healthy diet and increasing physical activity among women with APOs, starting in the postpartum setting.

**Mapping to PCR: Provides a downstream rationale for considering APO prevention within primary CVD prevention for women.**

**Quality:** Level I; AHA; peer-reviewed Circulation.

**[A8] Mehta, L. S., Warnes, C. A., Bradley, E., Burton, T., Economy, K., Mehran, R., Safdar, B., Sharma, G., Wood, M., Valente, A. M., & Volgman, A. S. (2020).** Cardiovascular Considerations in Caring for Pregnant Patients: A Scientific Statement From the American Heart Association. *Circulation*, 141(23), e884–e903. doi:10.1161/CIR.0000000000000772

*Evidence Level: I (AHA Scientific Statement)*

**Key findings:** Declares cardio-obstetrics as an essential multidisciplinary field. Covers HDP, MI, cardiomyopathies, arrhythmias, VHD, thromboembolism. Emphasizes preconception counseling by the multidisciplinary cardio-obstetrics team and team involvement through delivery and 1 year postpartum. Notes that CVD is the primary cause of pregnancy-related mortality in the United States.

**Mapping to PCR:** Foundational document for the cardio-obstetrics field within which PCR operates. Defines the pregnancy heart team (PHT).

**Quality:** Level I; AHA; peer-reviewed Circulation.

[A9] De Backer, J., Haugaa, K. H., Eide Hasselberg, N., de Hosson, M., Brida, M., et al. (2025). 2025 ESC Guidelines for the management of cardiovascular disease and pregnancy. European Heart Journal, 46, 4462-4568. doi:10.1093/eurheartj/ehaf193

Evidence Level: I (ESC Guideline)

Key findings: The guideline emphasizes pre-pregnancy counselling, individualized risk stratification, Pregnancy Heart Team care, and follow-up from preconception to the late postpartum period.

Mapping to PCR: Defines the guideline-based pathway that PCR could complement upstream; PCR does not replace mWHO classification, lesion-specific counselling, or contraindication-based advice.

Quality: Professional society guideline; included for framework alignment rather than as evidence of PCR efficacy.

**CATEGORY B: NARRATIVE EVIDENCE-MAPPING CONTEXT (1 source)**

**[B1] Padhani, Z. A., Rahim, K. A., Avery, J. C., Tessema, G. A., Castleton, P., Nisa, S., Damabi, N. M., Boyle, J. A., Salam, R. A., Meherali, S., & Lassi, Z. S. (2025).** Preconception Care Interventions Among Adolescents and Young Adults to Prevent Adverse Maternal, Perinatal and Child Health Outcomes: An Evidence Gap Map. *Public Health*, 239, 37–47. doi:10.1016/j.puhe.2024.12.036

*Evidence Level: I (Evidence Gap Map / Systematic)*

**Key findings:** Campbell Collaboration EGM. 18 studies (124 papers). Most evidence from higher-income countries; limited evidence on males/partners. Major gaps: behavioral interventions, community/school/digital delivery, non-clinical settings. Most frequent outcomes: perinatal > maternal > child. Quality of SRs generally moderate; most trials had "some concerns" (RoB 2).

**Mapping to PCR: Provides contextual methodology for organizing intervention, outcome, and phase domains. Its formal EGM workflow was not applied to the present purposive narrative evidence map.**

**Quality:** Level I; peer-reviewed Public Health; Open Access.

**CATEGORY C: CARDIO-OBSTETRICS (7 sources)**

**[C1] Vanharen, Y., Stocker, L., Mackillop, L., Van Berendoncks, A., Mannaerts, D., Goossens, E., Mulders, A. M., Kauling, R. M., & Schoenaker, D. (2026).** A Blueprint of an Integrated Cardio-Obstetric Preconception Care Pathway for Women With Pre-Pregnancy Intermediate- to High-Risk Cardiovascular Disease. *European Journal of Obstetrics, Gynecology, and Reproductive Biology*, 324, 115213. doi:10.1016/j.ejogrb.2026.115213

*Evidence Level: V (Review / Pathway Blueprint)*

**Key findings:** Most recent (2026). Three-step pathway: (1) comprehensive assessment + mWHO 2.0 risk stratification; (2) person-centered interdisciplinary counseling; (3) health optimization (medical, lifestyle, comorbidity, genetic). First structured preconception care pathway for mWHO Class II–III to IV women. Calls for implementation science research.

**Mapping to PCR:** Directly supports PCR Phase 1 (assessment) and Phase 2 (intervention planning). PCR extends this blueprint by adding CR-specific exercise prescription and program quality components.

**Quality:** Level V; peer-reviewed Eur J Obstet Gynecol Reprod Biol; very high currency.

**[C3] Misra, A., Porras, M. P., Rajendran, A., Contreras, J., & Scott, N. S. (2023).** Cardio-Obstetrics: A Focused Review. *Current Cardiology Reports*, 25(9), 1065–1073. doi:10.1007/s11886-023-01928-0

*Evidence Level: V (Review)*

**Key findings:** Covers preconception counseling, high-risk conditions, risk scores (CARPREG II, ZAHARA, mWHO), and multidisciplinary team management.

**Mapping to PCR:** Risk stratification tools (mWHO, CARPREG II, ZAHARA) integrated into PCR Phase 1 assessment.

**Quality:** Level V; peer-reviewed Curr Cardiol Rep.

**[C6] Sharma, G., Ying, W., & Silversides, C. K. (2021).** The Importance of Cardiovascular Risk Assessment and Pregnancy Heart Team in the Management of Cardiovascular Disease in Pregnancy. *Cardiology Clinics*, 39(1), 7–19. doi:10.1016/j.ccl.2020.09.002

*Evidence Level: V (Review)*

**Key findings:** Defines the Pregnancy Heart Team: preconception counseling, risk determination, antenatal/delivery plan, postpartum follow-up.

**Mapping to PCR:** Defines the team structure within which PCR operates.

**Quality:** Level V; peer-reviewed Cardiol Clin.

**[C7] Levine, L. D., Klammer, C., & Lewey, J. (2026).** Cardiovascular-Related Maternal Mortality. *Obstetrics and Gynecology*, 147(6), 796–805. doi:10.1097/AOG.0000000000006297

*Evidence Level: IV (Epidemiological)*

**Key findings:** CVD is the leading cause of pregnancy-related death in the US and disproportionately affects Black women. 68% of pregnancy-related CVD deaths are potentially preventable. Reviews evidence-based approaches to cardiovascular risk management in reproductive-aged women. Discusses the implementation of standardized care such as perinatal CVD risk assessment algorithms and postpartum remote monitoring programs.

**Mapping to PCR: Supports the rationale for earlier cardiovascular risk recognition and preconception intervention research.**

**Quality:** Level IV; peer-reviewed Obstet Gynecol; highest currency (2026).

**[C8] Goldberg, R. L., Prabakaran, S., & Scott, N. S. (2025).** Cardio Obstetrics: Bridging Heart and Pregnancy Health. *Trends in Cardiovascular Medicine*, 35(3), 166–174. doi:10.1016/j.tcm.2024.10.005

*Evidence Level: V (Review)*

**Key findings:** Most cardiovascular clinicians lack formal cardio-obstetric training. Substantial knowledge and care gaps exist. Reviews guiding principles for evaluation and management of pregnancy-capable individuals.

**Mapping to PCR:** Identifies the training and knowledge gap that PCR could help fill. Supports the need for standardized preconception cardiovascular care.

**Quality:** Level V; peer-reviewed Trends Cardiovasc Med.

**[C9] Davis, M. B., Arendt, K., Bello, N. A., Brown, H., Briller, J., Epps, K., Hollier, L., Langen, E., Park, K., Walsh, M. N., Williams, D., Wood, M., Silversides, C. K., & Lindley, K. J. (2021).** Team-Based Care of Women With Cardiovascular Disease From Pre-Conception Through Pregnancy and Postpartum. *Journal of the American College of Cardiology*, 77(14), 1763–1777. doi:10.1016/j.jacc.2021.02.033

*Evidence Level: V (JACC Focus Seminar)*

**Key findings:** Multidisciplinary cardio-obstetrics team approach: preconception risk assessment → antenatal monitoring → labor/delivery planning → fourth-trimester follow-up. Long-term CVD risk for women with APOs highlighted.

**Mapping to PCR:** The multidisciplinary team model within which PCR would operate. PCR adds the rehabilitation and exercise dimension to this team.

**Quality:** Level V; JACC Focus Seminar; high credibility.

**[C10] O'Kelly, A. C., Scott, N., & DeFaria Yeh, D. (2021).** Delivering Coordinated Cardio-Obstetric Care From Preconception Through Postpartum. *Cardiology Clinics*, 39(1), 163–173. doi:10.1016/j.ccl.2020.09.012

*Evidence Level: V (Review)*

**Key findings:** Detailed framework for coordinated care across the entire reproductive timeline. Addresses maternal mortality driven by increasing CVD burden during pregnancy. Emphasizes that coordinated preconception through postpartum cardio-obstetrics care is necessary to optimize both maternal and fetal health.

**Mapping to PCR:** Coordinated care framework that PCR would integrate into.

**Quality:** Level V; peer-reviewed Cardiol Clin.

**CATEGORY D: PRECONCEPTION INTERVENTION EVIDENCE (10 sources)**

**[D1] Poprzeczny, A. J., Mitchell, M., Deussen, A. R., Habibi, N., Cheung, T. O., & Grieger, J. A. (2025).** Pre-Pregnancy Diet and/or Physical Activity Interventions for the Prevention of Gestational Diabetes Mellitus: A Systematic Review and Meta-Analysis. *Australian and New Zealand Journal of Obstetrics and Gynaecology*, 65(6), 719–728. doi:10.1111/ajo.70032

*Evidence Level: I (SR/MA)*

**Key findings:** 5 RCTs (n = 1,924). No significant reduction in GDM (RR 1.19, 95% CI 0.81–1.75). No significant differences in HDP, PTB, cesarean delivery, or GWG. Significant heterogeneity between studies in type, duration, and intensity of interventions. Key gap: no long-term maternal or child outcomes.

**Mapping to PCR: Highlights limited direct trial evidence. The cited RCTs did not evaluate integrated PCR or establish the effectiveness of CR-level preconception interventions.**

**Quality:** Level I; peer-reviewed Aust N Z J Obstet Gynaecol.

**[D3] Al-Huda, F., Shapiro, G. D., Davenport, M. H., Bertagnolli, M., & Dayan, N. (2022).** Association Between Cardiorespiratory Fitness and Hypertensive Disorders of Pregnancy: A Systematic Review and Meta-Analysis. *Journal of Clinical Medicine*, 11(15), 4364. doi:10.3390/jcm11154364

*Evidence Level: I (SR/MA)*

**Key findings:** 14 studies (n = 2,406). Lower preconception CRF (VO2max < 37 mL/kg/min) associated with increased HDP risk. VO2max at 14–18 weeks marginally lower in women who later developed PE (mean difference 0.43 mL/kg/min). Postpartum VO2peak trended lower in women with prior PE. CRF may identify at-risk women and HDP may confer a hit to cardiorespiratory reserve.

**Mapping to PCR:** Direct evidence for PCR Phase 1 (CPET assessment). CRF as a modifiable risk factor → target for PCR exercise training.

**Quality:** Level I; peer-reviewed J Clin Med; indexed in WoS.

**[D4] Murrell, K. S. & Joseph, R. P. (2025).** Preconception Physical Activity and Maternal Health Outcomes: An Integrative Review of Studies from 2016–2025. *Maternal and Child Health Journal*, 29(7), 857–869. doi:10.1007/s10995-025-04086-7

*Evidence Level: I (Integrative Review)*

**Key findings:** Only 7 articles met inclusion criteria from 3,608 screened. Higher preconception MVPA associated with reduced gestational hypertension, PE, GDM, excessive GWG, and LBW. Greater risk reduction with higher MVPA levels.

**Mapping to PCR:** Direct evidence for PCR Phase 2 (exercise prescription). Quantifies the protective association of preconception physical activity.

**Quality:** Level I; peer-reviewed Matern Child Health J; high currency (2025).

**[D6] Hayes, L., McParlin, C., Azevedo, L. B., Jones, D., Newham, J., Olajide, J., McCleman, L., & Heslehurst, N. (2021).** The Effectiveness of Smoking Cessation, Alcohol Reduction, Diet and Physical Activity Interventions in Improving Maternal and Infant Health Outcomes: A Systematic Review of Meta-Analyses. *Nutrients*, 13(3), 1036. doi:10.3390/nu13031036

*Evidence Level: I (Umbrella Review)*

**Key findings:** 65 SRs (602 meta-analyses). Consistent evidence for diet + PA interventions: reduced GWG, postnatal weight retention, cesarean delivery, PE, hypertension, GDM, and PTD. No effect on LBW, HBW, NICU admission, Apgar scores, or mortality.

**Mapping to PCR: Pregnancy-phase comparator evidence. It informs plausibility but does not substitute for preconception intervention evidence.**

**Quality:** Level I; peer-reviewed Nutrients; umbrella review of 65 SRs.

**[D7] Kibret, K. T., Chojenta, C., Gresham, E., Tegegne, T. K., & Loxton, D. (2019).** Maternal Dietary Patterns and Risk of Adverse Pregnancy (Hypertensive Disorders of Pregnancy and Gestational Diabetes Mellitus) and Birth (Preterm Birth and Low Birth Weight) Outcomes: A Systematic Review and Meta-Analysis. *Public Health Nutrition*, 22(3), 506–520. doi:10.1017/S1368980018002616

*Evidence Level: I (SR/MA)*

**Key findings:** 21 observational studies. Healthy dietary pattern (vegetables, fruits, legumes, whole grains) → PE OR 0.78 (95% CI 0.70–0.86), GDM OR 0.78 (0.56–0.99), PTB OR 0.75 (0.57–0.93).

**Mapping to PCR:** Supports PCR Phase 2 nutritional counseling component. Observational evidence for dietary pattern modification before pregnancy.

**Quality:** Level I; peer-reviewed Public Health Nutr.

**[D8] Suzuki, H., Tun, P. P., Liu, S., Ota, E., & Arata, N. (2025).** Effectiveness of Web-Based Interventions on Clinical Outcomes and Lifestyle Modifications in Women Planning to Conceive: A Systematic Review. *Healthcare*, 13(9), 1037. doi:10.3390/healthcare13091037

*Evidence Level: I (SR)*

**Key findings:** 4 RCTs (n = 1,965). Benefits observed for systolic blood pressure, serum folate levels, and physical activity. No significant effects on vegetable/fruit intake, folic acid supplementation, smoking, alcohol, waist circumference, weight, BMI, HbA1c, cholesterol, or pregnancy outcomes. PROSPERO CRD42023488277.

**Mapping to PCR: Informs delivery-model considerations; a hybrid rather than purely digital approach may warrant evaluation.**

**Quality:** Level I; peer-reviewed Healthcare; PROSPERO registered.

**[D9] Xiong, Y., Chen, J., Wu, Y., Zhao, P., Liao, M., Guo, J., Liu, C., Zheng, M., Ren, Y., Zou, K., Sun, X., & Tan, J. (2025).** The Effect of Maternal Pre-Pregnancy Body Mass Index on Hypertensive Disorders of Pregnancy (HDP): A Systematic Review and Dose-Response Meta-Analysis of Cohort Studies Involving 50 Million Pregnancies. *eClinicalMedicine*, 86, 103395. doi:10.1016/j.eclinm.2025.103395

*Evidence Level: I (SR/MA)*

**Key findings:** 157 cohort studies (n ≈ 51.81 million). BMI per 5 kg/m² increase → HDP aOR = 1.75. Asian populations show higher risk at equivalent BMI. Strongest dose-response meta-analysis to date on preconception BMI and HDP.

**Mapping to PCR: Identifies preconception BMI as a candidate target for PCR weight-management and nutrition components.**

**Quality: Level I; peer-reviewed eClinicalMedicine (Lancet family).**

**[D10] Wang, S., Mitsunami, M., Ortiz-Panozo, E., Leung, C. W., Manson, J. E., Rich-Edwards, J. W., & Chavarro, J. E. (2023).** Prepregnancy Healthy Lifestyle and Adverse Pregnancy Outcomes. *Obstetrics and Gynecology*, 142(6), 1278–1290. doi:10.1097/AOG.0000000000005346

*Evidence Level: IV (Prospective Cohort, NHSII)*

**Key findings:** 15,509 women, 27,135 pregnancies. Six low-risk lifestyle factors (healthy BMI, nonsmoking, ≥150 min/wk MVPA, healthy diet, no/low alcohol, multivitamin use) were inversely associated with APOs in a dose-dependent manner. Women with all six factors had 37% lower risk of APOs (RR 0.63, 95% CI 0.55–0.72). Population attributable risk: 19% of APOs could be prevented by adopting all six factors.

**Mapping to PCR: Prospective observational evidence supporting the rationale for multicomponent preconception lifestyle optimization; it does not evaluate integrated PCR as an intervention.**

**Quality:** Level IV; NHSII cohort; peer-reviewed Obstet Gynecol.

**[D11] Santos, S., Voerman, E., Amiano, P., Barros, H., Beilin, L. J., Bergström, A., ... & Jaddoe, V. W. V. (2019).** Impact of Maternal Body Mass Index and Gestational Weight Gain on Pregnancy Complications: An Individual Participant Data Meta-Analysis of European, North American and Australian Cohorts. *BJOG: An International Journal of Obstetrics & Gynaecology*, 126(8), 984–995. doi:10.1111/1471-0528.15661

*Evidence Level: I (IPD Meta-Analysis)*

**Key findings:** 39 cohorts, 265,270 births. Obese mothers with high GWG had highest risk of any pregnancy complication (OR 2.51, 95% CI 2.31–2.74). 23.9% of any pregnancy complication attributable to maternal overweight/obesity. Pre-pregnancy BMI and GWG are, across their full ranges, associated with risks of pregnancy complications.

**Mapping to PCR: Quantifies the population-attributable burden associated with preconception BMI and supports its consideration as a PCR target.**

**Quality:** Level I; peer-reviewed BJOG; individual participant data meta-analysis.

**[D12] Daly, M., Kipping, R. R., Tinner, L. E., Sanders, J., & White, J. W. (2022).** Preconception Exposures and Adverse Pregnancy, Birth and Postpartum Outcomes: Umbrella Review of Systematic Reviews. *Paediatric and Perinatal Epidemiology*, 36(2), 288–299. doi:10.1111/ppe.12855

*Evidence Level: I (Umbrella Review)*

**Key findings:** 53 SRs, 205 unique exposure-outcome associations. High-certainty evidence: maternal folate supplementation reduces neural tube defects. Moderate-certainty evidence: maternal physical activity associated with reduced risk of pre-eclampsia and GDM; maternal BMI and interpregnancy weight gain associated with increased risk. Methodological quality generally low — only 2 reviews rated "high" quality.

**Mapping to PCR:** Highest-level evidence synthesis of preconception exposures. Identifies maternal PA and BMI as the most evidence-supported modifiable preconception factors.

**Quality:** Level I; peer-reviewed Paediatr Perinat Epidemiol; GRADE-assessed.

**CATEGORY E: POSTPARTUM CARDIOVASCULAR (3 sources)**

**[E1] Lewey, J., Beckie, T. M., Brown, H. L., Brown, S. D., Garovic, V. D., Khan, S. S., Miller, E. C., Sharma, G., & Mehta, L. S. (2024).** Opportunities in the Postpartum Period to Reduce Cardiovascular Disease Risk After Adverse Pregnancy Outcomes: A Scientific Statement From the American Heart Association. *Circulation*. doi:10.1161/CIR.0000000000001212

*Evidence Level: I (AHA Scientific Statement)*

**Key findings:** The fourth trimester (12 weeks postpartum) is a critical window for cardiovascular health intervention. Reviews interpregnancy and postpartum preventive strategies, BP management, and lifestyle interventions aligned with LE8. Calls for clinical, health-system, and community-level interventions. Individuals with APOs have an increased incidence of CVD risk factors after delivery.

**Mapping to PCR:** Defines the postpartum bridge — PCR graduates return to this framework for ongoing cardiovascular risk management.

**Quality:** Level I; AHA; peer-reviewed Circulation.

**[E3] Staff, A. C., Costa, M. L., Dechend, R., Jacobsen, D. P., & Sugulle, M. (2024).** Hypertensive Disorders of Pregnancy and Long-Term Maternal Cardiovascular Risk: Bridging Epidemiological Knowledge Into Personalized Postpartum Care and Follow-Up. *Pregnancy Hypertension*, 36, 101127. doi:10.1016/j.preghy.2024.101127

*Evidence Level: V (Review)*

**Key findings:** Pregnancy serves as a "stress test" for future CVD. Modifiable factors underexploited postpartum: breastfeeding, BP management. Systematic cardio-preventive follow-up after HDP is recommended.

**Mapping to PCR:** The "stress test" metaphor supports PCR's preconception cardiovascular optimization approach.

**Quality:** Level V; peer-reviewed Pregnancy Hypertens.

**[E6] Moholdt, T., Garnæs, K. K., Vik, I. P., Mørkved, S., Salvesen, K. Å., & Ingul, C. B. (2024).** Cardiovascular Effects of Exercise Training in Pregnant People With a High Body Mass Index: Secondary Results From a Randomised Controlled Trial (ETIP). *BMJ Open Sport & Exercise Medicine*, 10(4), e002099. doi:10.1136/bmjsem-2024-002099

*Evidence Level: II (RCT, secondary analysis)*

**Key findings:** 87 participants (BMI ≥28 kg/m²). Exercise training during pregnancy: no statistically significant effect on VO2peak at 34–37 weeks (1.7 mL/min/kg, 95% CI −0.4 to 3.7) or postpartum. No significant between-group differences in FMD or echocardiographic outcomes. Only 50% fulfilled prespecified adherence criteria. Norwegian single-center RCT (NCT01243554).

**Mapping to PCR: Pregnancy-stage evidence that informs plausibility but does not establish that preconception exercise would produce larger effects. Adherence remains an implementation challenge.**

**Quality:** Level II; peer-reviewed BMJ Open SEM; RCT evidence.

**CATEGORY F: MECHANISMS / PHYSIOLOGY (7 sources)**

**[F1] Bernstein, I. M., Badger, G. J., & McBride, C. A. (2025).** Prepregnancy Physiology and Subsequent Preterm Preeclampsia. *American Journal of Obstetrics and Gynecology*, 232(3), 314.e1–314.e12. doi:10.1016/j.ajog.2024.05.031

*Evidence Level: IV (Prospective Cohort)*

**Key findings:** 124 participants (81 nulliparous, 43 with prior preterm PE). Women who developed preterm PE had greater android fat (3,215 vs 1,918 g), faster supine pulse (77 vs 67 bpm), higher diastolic BP (82 vs 68 mmHg), increased cardiac output (5.6 vs 4.6 L/min), faster aortic-popliteal PWV (4.5 vs 3.8 m/s), higher CRP (10.7 vs 4.1 mg/mL), and greater insulin resistance (HOMA-IR 2.2 vs 1.2). Factors consistent with subclinical metabolic syndrome are detectable before pregnancy.

**Mapping to PCR: Mechanistic evidence relevant to PCR Phase 1. Prepregnancy physiological parameters may inform cardiovascular assessment and risk stratification.**

**Quality:** Level IV; peer-reviewed Am J Obstet Gynecol.

**[F2] Gyselaers, W. & Lees, C. (2022).** Maternal Low Volume Circulation Relates to Normotensive and Preeclamptic Fetal Growth Restriction. *Frontiers in Medicine*, 9, 902634. doi:10.3389/fmed.2022.902634

*Evidence Level: V (Narrative Review)*

**Key findings:** Low intravascular volume may explain why high vascular resistance causes hypertension in PE with FGR while presenting with normotension in FGR alone. Poor plasma volume expandability may already be present before conception. Experimentally induced low plasma volume in rodents predisposes to poor fetal growth. Proposes elevating maternal intravascular volume with physical exercise ± Nitric Oxide Donors in FGR and PE.

**Mapping to PCR: Mechanistic and indirect evidence linking maternal cardiovascular status with fetal growth. It supports biological plausibility but not a direct PCR effect on fetal growth restriction.**

**Quality:** Level V; peer-reviewed Front Med; indexed in PMC.

**[F3] Foo, F. L. (2019).** A Prospective Longitudinal Study of Maternal Cardiovascular Function From Pre-Conception to Post-Partum: The CONCEIVE Study (Doctoral dissertation). Imperial College London. doi:10.25560/75097

*Evidence Level: IV (Prospective Cohort)*

**Key findings:** 387 women tracked from preconception through postpartum. Women who later developed PE or FGR exhibited a distinct hemodynamic phenotype before pregnancy — lower cardiac output, higher systemic vascular resistance. Subclinical cardiovascular dysfunction is detectable and potentially modifiable before conception. Pre-pregnancy exercise stress testing may help distinguish women at risk.

**Mapping to PCR: Prospective mechanistic evidence indicating that a hemodynamic phenotype may precede preeclampsia or fetal growth restriction. It supports the rationale for assessment, not the efficacy of PCR optimization.**

**Quality:** Level IV; Imperial College London; prospective longitudinal design.

**[F4] Zhuang, Y., Li, L., Zhang, Y., Liu, X., Zeng, B., Zhu, B., & Dai, F. (2025).** Association Between Life's Essential 8 and Infertility as Well as the Mediating Effects of Oxidative Stress and Inflammatory Factors Among U.S. Women Aged 18–45 Years. *Reproductive Sciences*, 32(3), 738–747. doi:10.1007/s43032-024-01635-3

*Evidence Level: IV (Cross-sectional / Population)*

**Key findings:** LE8 framework validated in reproductive-age women. Oxidative stress and inflammatory factors mediate the LE8–infertility link. Demonstrates LE8 relevance to the reproductive-age population.

**Mapping to PCR:** LE8 as a measurement framework for PCR outcomes. Supports the applicability of the AHA CVH metric to preconception populations.

**Quality:** Level IV; peer-reviewed Reprod Sci; PubMed indexed.

**[F5] Lihme, F., Basit, S., Persson, L. G., Larsen, M. O., Lauridsen, K. H., Lykke, J. A., Andersen, A. S., Thorsen-Meyer, A., Pihl, K., Melbye, M., Wohlfahrt, J., & Boyd, H. A. (2024).** Third-Trimester Cardiovascular Function and Risk of Hypertensive Disorders of Pregnancy. *Journal of the American Heart Association*, 13(20), e032673. doi:10.1161/JAHA.123.032673

*Evidence Level: IV (Case-Cohort)*

**Key findings:** 906 pregnant women in Denmark. Low CO strongly associated with preeclampsia at <37 weeks (with FGR: HR 5.25, 95% CI 1.26–21.9; without FGR: HR 2.19, 95% CI 1.07–4.48). High systemic vascular resistance strongly associated with increased rates of all HDP forms. Third-trimester drop in CO >75th percentile associated with preeclampsia in women without FGR (OR 1.91).

**Mapping to PCR:** Demonstrates that low CO and high SVR are associated with PE regardless of FGR status. Complements F3 (CONCEIVE) by providing third-trimester hemodynamic data.

**Quality:** Level IV; peer-reviewed J Am Heart Assoc; case-cohort design.

**[F6] Skow, R. J., King, E. C., Steinback, C. D., & Davenport, M. H. (2017).** The Influence of Prenatal Exercise and Pre-Eclampsia on Maternal Vascular Function. *Clinical Science*, 131(17), 2223–2240. doi:10.1042/CS20171036

*Evidence Level: V (Review)*

**Key findings:** Healthy pregnancy involves arterial remodeling (increased angiogenesis, vasodilation), reduced arterial stiffness, and enhanced endothelial function. PE is associated with poor placentation, increased arterial stiffness, and vascular dysfunction. Prenatal exercise enhances placental angiogenesis and endothelial function. Maternal exercise may reduce the risk of vascular dysfunction in pregnancy.

**Mapping to PCR: Provides a mechanistic rationale for examining whether exercise may support vascular adaptation in a future PCR programme.**

**Quality:** Level V; peer-reviewed Clin Sci.

**[F7] Rodríguez, I. & González, M. (2014).** Physiological Mechanisms of Vascular Response Induced by Shear Stress and Effect of Exercise in Systemic and Placental Circulation. *Frontiers in Pharmacology*, 5, 209. doi:10.3389/fphar.2014.00209

*Evidence Level: V (Review)*

**Key findings:** Shear stress stimulates eNOS-NO pathway, promoting vasodilation and angiogenesis. Exercise-induced shear stress upregulates VEGF and eNOS expression, potentially improving placental perfusion. Provides the molecular framework for exercise as a placental-protective intervention.

**Mapping to PCR: Provides a mechanistic rationale linking exercise, shear stress, and placental vascular function.**

**Quality:** Level V; peer-reviewed Front Pharmacol.

**CATEGORY G: ADDITIONAL (1 source)**

**[G1] Torkel, S., Mantzioris, E., Villani, A., Kellow, N. J., Bhatnagar, D., Osei-Safo, E. K., McGowan, M., Abdul Jafar, N. K., Bogatzke, N., Alesi, S., Astarcioglu, T., Mol, B. W., Norman, R. J., Cowan, S., Wang, R., & Moran, L. (2026).** Preconception Lifestyle Interventions for Women — A Systematic Review and Meta-Analysis of Intervention Characteristics and Behaviour Change Techniques. *Human Reproduction Update*, 32(1), 105–127. doi:10.1093/humupd/dmaf021

*Evidence Level: I (SR/MA)*

**Key findings:** 24 RCTs (n = 7,795). No overall difference in clinical pregnancy (OR 1.06, 95% CI 0.84–1.35) or live birth (OR 1.17, 0.82–1.67). However, ≥10 sessions increased clinical pregnancy odds (OR 2.17, 95% CI 1.21–3.86). Adding objects to the environment (e.g., provision of food/exercise equipment) increased clinical pregnancy (OR 3.51, 95% CI 1.70–7.23). Significant weight reduction (−3.87 kg) and FBG improvement (−0.15 mM). Face-to-face delivery superior for weight loss (−6.02 kg). PROSPERO CRD42022333066.

**Mapping to PCR:** Identifies effective behavior change techniques for PCR intervention design. Face-to-face delivery with ≥10 sessions and provision of equipment may be optimal. BCT taxonomy v1 provides a framework for PCR behavior change strategy.

**Quality:** Level I; peer-reviewed Hum Reprod Update (IF ~15); top-tier journal.

## CATEGORY H: IMPLEMENTATION SCIENCE (3 sources)

[H1] Proctor, E., Silmere, H., Raghavan, R., Hovmand, P., Aarons, G., Bunger, A., Griffey, R., & Hensley, M. (2011). Outcomes for implementation research: conceptual distinctions, measurement challenges, and research agenda. Administration and Policy in Mental Health, 38, 65-76. doi:10.1007/s10488-010-0319-7

Evidence Level: V (Conceptual implementation framework)

Mapping to PCR: Provides implementation outcomes, including acceptability, adoption, appropriateness, feasibility, fidelity, cost, penetration, and sustainability, for future PCR evaluation.

[H2] Glasgow, R. E., Vogt, T. M., & Boles, S. M. (1999). Evaluating the public health impact of health promotion interventions: the RE-AIM framework. American Journal of Public Health, 89, 1322-1327. doi:10.2105/AJPH.89.9.1322

Evidence Level: V (Implementation framework)

Mapping to PCR: Provides a framework for evaluating reach and maintenance alongside effectiveness, adoption, and implementation.

[H3] Damschroder, L. J., Reardon, C. M., Widerquist, M. A. O., & Lowery, J. (2022). The updated Consolidated Framework for Implementation Research based on user feedback. Implementation Science, 17, 75. doi:10.1186/s13012-022-01245-0

Evidence Level: V (Implementation framework)

Mapping to PCR: Provides a determinant framework for examining referral, staffing, adherence, financing, and health-system integration.

**Supplementary File 4. Source Verification Report**

**1. Evidence Level Distribution**

| Level | Type | Count | Sources |
| --- | --- | --- | --- |
| I | SR/MA / Scientific Statements / EGM / Umbrella Review / IPD Meta-Analysis | 17 | A1, A2, A6, A7, A8, B1, D1, D3, D4, D6, D7, D8, D9, D11, D12, E1, G1 |
| II | RCT | 1 | E6 |
| III | Controlled non-randomized | 0 | — |
| IV | Cohort / Case-Control / Cross-sectional | 6 | C7, D10, F1, F3, F4, F5 |
| V | Reviews / Pathway Blueprints / Narrative Reviews | 11 | A5, C1, C3, C6, C8, C9, C10, E3, F2, F6, F7 |

**Key: 49% of sources are Level I by study-design descriptor, mainly AHA scientific statements and systematic reviews. This distribution supports framework development but does not constitute a formal certainty assessment.**

**2. Predatory Journal Screen**

| Source | Journal | Indexed? | Suspicious? | Verdict |
| --- | --- | --- | --- | --- |
| A1 | Circulation | WoS/Scopus/PubMed | No | CLEAN |
| A2 | Circulation | WoS/Scopus/PubMed | No | CLEAN |
| A5 | Front Cardiovasc Med | WoS/Scopus/PubMed | No | CLEAN |
| B1 | Public Health | WoS/Scopus/PubMed | No | CLEAN |
| C1 | Eur J Obstet Gynecol Reprod Biol | WoS/Scopus/PubMed | No | CLEAN |
| C7 | Obstet Gynecol | WoS/Scopus/PubMed | No | CLEAN |
| D9 | eClinicalMedicine | WoS/Scopus/PubMed | No | CLEAN |
| G1 | Hum Reprod Update | WoS/Scopus/PubMed (IF ~15) | No | CLEAN |
| All others | Various | All WoS/Scopus/PubMed indexed | No | CLEAN |

**Result: No predatory journals or preprints were identified in the curated source set.**

**3. Conflict of Interest (COI) Assessment**

| Concern Level | Count | Description |
| --- | --- | --- |
| NONE | 33 | AHA/ESC statements, academic SRs — standard academic funding |
| LOW | 2 | A5 (exercise physiology — possible fitness industry links, not declared); C1 (pharma funding not excluded, but no specific COI noted) |
| MODERATE | 0 | — |
| HIGH | 0 | — |

**Action:** No critical COI concerns. Standard academic funding across all sources.

**4. Currency Assessment**

| Period | Count | Sources |
| --- | --- | --- |
| 2014 | 1 | F7 (foundational mechanistic review — timeless) |
| 2017 | 1 | F6 (mechanism review — still relevant) |
| 2019 | 3 | D7, D11, F3 (F3 = landmark CONCEIVE study; D11 = foundational IPD meta-analysis) |
| 2020 | 1 | A8 (AHA foundational — still current) |
| 2021 | 5 | A7, C6, C9, C10, D6 |
| 2022 | 3 | D3, D12, F2 |
| 2023 | 3 | A2, C3, D10 |
| 2024 | 6 | A1, A5, E1, E3, E6, F5 |
| 2025 | 8 | A6, B1, D1, D4, D8, D9, F1, F4 |
| 2026 | 4 | C1, C7, C8, G1 |

**Currency score:** 18/35 (51%) from 2024–2026. The source base spans foundational mechanistic work (2014–2019) and recent clinical and framework literature (2020–2026), reflecting the interdisciplinary nature of the field.

**5. Critical Fact-Check: Key Claims Verified**

**Claim: "68% of pregnancy-related CVD deaths are preventable"**

- Source: Levine et al. (2026, *Obstet Gynecol*) [C7]

- Cross-check: AHA 2023 (A2); Mehta 2020 (A8)

- Verdict: CONFIRMED — consistent across 3 independent AHA sources.

**Claim: "Only 5 preconception RCTs exist" (Poprzeczny 2025)**

- Source: Poprzeczny et al. (2025) [D1]

- Cross-check: Murrell (2025) [D4] found 7 studies total (not all RCTs); G1 Torkel (2026) found 24 RCTs in infertility populations (different population)

- Verdict: CONSISTENT for general preconception populations — the 5-RCT figure is specific to diet/PA interventions for GDM prevention in general preconception populations.

**Claim: "No identified study evaluated integrated preconception cardiac rehabilitation"**

- Source: Curated narrative evidence corpus and structured database searches

- Cross-check: No integrated PCR intervention was identified in the curated corpus; searches were not retained as an exhaustive, deduplicated screening dataset.

- Verdict: No direct integrated PCR evaluation was identified. This is a framework-level evidence gap, not an exhaustive systematic-search conclusion.

**Claim: "VO2max < 37 mL/kg/min preconception → increased HDP risk"**

- Source: Al-Huda et al. (2022) [D3]

- Cross-check: Bernstein et al. (2025) [F1] — prepregnancy hemodynamic differences predict PE; Foo (2019) [F3] — lower CO/higher SVR phenotype before conception

- Verdict: LIKELY ACCURATE — consistent across multiple study designs, though based on a small number of primary studies; treat as suggestive rather than a definitive threshold.

**6. Source Verification Overall Assessment**

| Metric | Result |
| --- | --- |
| Total sources verified | 35 |
| Confirmed legitimate (no issues) | 35 (100%) |
| Preprint (conditional) | 0 |
| Pending COI check | 0 |
| Predatory / Retracted | 0 |
| Evidence Level distribution | 49% Level I; no Level VI/VII |
| Currency (2024–2026) | 51% |
| VERDICT | PASS — Source base is high-quality, current, and appropriate for a structured narrative review with EGM |

**7. Gaps in Source Coverage**

- No Chinese-language empirical studies (CNKI search returned 0 on topic) — this is itself a finding (Chinese literature gap).

- No qualitative or mixed-methods studies on women's experiences of preconception cardiovascular interventions.

- No health-economic or cost-effectiveness analyses.

- Limited LMIC evidence — most sources are from high-income countries (US, UK, Europe, Australia).

- No male partner preconception cardiovascular health studies.

- Limited FGR-focused preconception intervention evidence — the mechanistic link (F2, F3, F5) is stronger than the intervention evidence.

**Supplementary File 5. Reference-Manager Library**

The reference-manager library should be regenerated from the final curated source set of 39 sources, including the ESC guideline and the three implementation-science sources added in this revision. Before submission, authors should verify that the exported library and manuscript reference list contain the same final records.

**Supplementary File 6. Per-Source Evidence Classification**

This table provides the study design, evidence descriptor, domain relevance, and appraisal notes for each of the 39 curated sources. Evidence descriptors should not be interpreted as formal certainty ratings.

| ID | First Author (Year) | Study Design | Evidence Level | Domain Relevance | Key Appraisal Notes |
| --- | --- | --- | --- | --- | --- |
| A1 | Brown (2024) | AHA Scientific Statement | I | CR core components | 2024 AHA/AACVPR update; 9 components; primary PCR framework source |
| A2 | Khan (2023) | AHA Scientific Statement | I | Preconception CVH | LE8 framework; life-course and intergenerational approach; calls for preconception intervention trials |
| A5 | Milani (2024) | Narrative Review | V | Exercise prescription | FITT-VP framework; CPET/ventilatory threshold guidance; practical low-resource alternatives |
| A6 | Mauricio (2025) | AHA Scientific Statement | I | ART population | CV risk assessment and counseling for ART patients; relevant PCR sub-population |
| A7 | Parikh (2021) | AHA Scientific Statement | I | APO–CVD link | Pregnancy as window to future CV health; downstream PCR rationale |
| A8 | Mehta (2020) | AHA Scientific Statement | I | Cardio-obstetrics | Defines pregnancy heart team; preconception counseling emphasis; foundational for field |
| B1 | Padhani (2025) | Evidence Gap Map | I | EGM methodology | Campbell Collaboration EGM; 18 studies; primary methodology template for PCR-EGM |
| C1 | Vanharen (2026) | Pathway Blueprint | V | Cardio-obstetrics | Three-step pathway; mWHO 2.0; first structured preconception care pathway for mWHO II–IV |
| C3 | Misra (2023) | Narrative Review | V | Cardio-obstetrics | Risk stratification tools (CARPREG II, ZAHARA, mWHO); preconception counseling |
| C6 | Sharma (2021) | Narrative Review | V | Cardio-obstetrics | Pregnancy Heart Team definition; risk assessment framework |
| C7 | Levine (2026) | Epidemiological Review | IV | Maternal mortality | 68% of CVD deaths preventable; evidence-based risk management approaches; critical PCR justification |
| C8 | Goldberg (2025) | Narrative Review | V | Cardio-obstetrics | Training/knowledge gaps; guiding principles for pregnancy-capable individuals |
| C9 | Davis (2021) | JACC Focus Seminar | V | Cardio-obstetrics | Multidisciplinary team model; preconception through postpartum; 14-author JACC series |
| C10 | O'Kelly (2021) | Narrative Review | V | Cardio-obstetrics | Coordinated care framework across reproductive timeline |
| D1 | Poprzeczny (2025) | SR/MA | I | Preconception intervention | 5 RCTs; null GDM results; primary "evidence cliff" source |
| D3 | Al-Huda (2022) | SR/MA | I | CRF–HDP association | 14 studies; VO2max < 37 mL/kg/min → HDP risk; direct PCR Phase 1 evidence |
| D4 | Murrell (2025) | Integrative Review | I | Preconception PA | 7 studies from 3,608 screened; preconception MVPA protective for multiple APOs |
| D6 | Hayes (2021) | Umbrella Review | I | Pregnancy interventions | 65 SRs (602 meta-analyses); pregnancy-phase evidence baseline for EGM comparison |
| D7 | Kibret (2019) | SR/MA | I | Dietary patterns | 21 observational studies; healthy diet → reduced PE, GDM, PTB |
| D8 | Suzuki (2025) | SR | I | Digital health | 4 RCTs (n = 1,965); web-based interventions mixed/null; PROSPERO registered |
| D9 | Xiong (2025) | SR/MA (Dose-Response) | I | BMI–HDP | 157 cohorts (51.81 million); BMI per 5 kg/m² → HDP aOR 1.75; Asian populations higher risk |
| D10 | Wang (2023) | Prospective Cohort (NHSII) | IV | Healthy lifestyle | 15,509 women; 6 healthy factors → 37% lower APO risk; PAR 19% |
| D11 | Santos (2019) | IPD Meta-Analysis | I | BMI/GWG | 39 cohorts (265,270 births); 23.9% APOs attributable to overweight/obesity |
| D12 | Daly (2022) | Umbrella Review | I | Preconception exposures | 53 SRs; moderate-certainty evidence for PA, BMI, interpregnancy weight gain |
| E1 | Lewey (2024) | AHA Scientific Statement | I | Postpartum CV | Fourth trimester as critical window; postpartum bridge for PCR; LE8-aligned interventions |
| E3 | Staff (2024) | Narrative Review | V | HDP–CVD link | Pregnancy as CV stress test; personalized postpartum follow-up framework |
| E6 | Moholdt (2024) | RCT (secondary) | II | Pregnancy exercise | ETIP trial (n = 87); BMI ≥28; exercise did not significantly improve VO2peak; adherence 50% |
| F1 | Bernstein (2025) | Prospective Cohort | IV | Prepregnancy physiology | 124 women; subclinical metabolic syndrome features predict preterm PE before conception |
| F2 | Gyselaers (2022) | Narrative Review | V | CV–FGR mechanism | Low volume circulation → FGR; proposes exercise + NO donors for plasma volume expansion |
| F3 | Foo (2019) | Prospective Cohort (Doctoral) | IV | CV function trajectory | CONCEIVE Study (n = 387); PE/FGR women show low CO/high SVR before conception |
| F4 | Zhuang (2025) | Cross-sectional | IV | LE8 validation | LE8 in reproductive-age women; oxidative stress/inflammation mediation |
| F5 | Lihme (2024) | Case-Cohort | IV | Hemodynamics–HDP | PEACH (n = 906); low CO → preterm PE HR 5.25 (with FGR); high SVR → all HDP |
| F6 | Skow (2017) | Narrative Review | V | Vascular mechanisms | Exercise enhances endothelial function, placental angiogenesis, arterial compliance |
| F7 | Rodríguez (2014) | Narrative Review | V | Shear stress mechanism | Shear stress–eNOS–NO pathway; exercise → VEGF/eNOS → placental perfusion |
| G1 | Torkel (2026) | SR/MA | I | Lifestyle interventions | 24 RCTs (n = 7,795); ≥10 sessions + equipment provision improve outcomes; BCT taxonomy v1 |
| A9 | De Backer (2025) | ESC guideline | I | Pre-pregnancy counselling; Pregnancy Heart Team | Guideline alignment; not evidence of PCR efficacy |
| H1 | Proctor (2011) | Implementation framework | V | Implementation outcomes | Defines acceptability, adoption, feasibility, fidelity, cost, penetration, sustainability |
| H2 | Glasgow (1999) | Implementation framework | V | Reach and maintenance | RE-AIM framework for future PCR evaluation |
| H3 | Damschroder (2022) | Implementation framework | V | Implementation determinants | CFIR framework for referral and health-system determinants |

**Notes:** Evidence levels follow the five-tier classification described in Supplementary File 2 Section 3. Domain relevance is assigned based on the CR core component(s) most directly addressed. Appraisal notes summarize the source's primary contribution to the PCR framework; see Supplementary File 3 for full annotation.
